# Insights into the structural and functional activities of forgotten Kinases: PCTAIREs CDKs

**DOI:** 10.1186/s12943-024-02043-6

**Published:** 2024-06-29

**Authors:** Javad Karimbayli, Ilenia Pellarin, Barbara Belletti, Gustavo Baldassarre

**Affiliations:** Division of Molecular Oncology, Centro di Riferimento Oncologico (CRO) of Aviano, IRCCS, National Cancer Institute, Via Franco Gallini, Aviano, 33081 Italy

**Keywords:** Atypical cyclin dependent kinases, atypical CDKs, CDK16, CDK17, CDK18, 3D structure, Small molecule CDK inhibitors, PCTAIREs in cancer

## Abstract

In cells, signal transduction heavily relies on the intricate regulation of protein kinases, which provide the fundamental framework for modulating most signaling pathways. Dysregulation of kinase activity has been implicated in numerous pathological conditions, particularly in cancer. The druggable nature of most kinases positions them into a focal point during the process of drug development. However, a significant challenge persists, as the role and biological function of nearly one third of human kinases remains largely unknown.

Within this diverse landscape, cyclin-dependent kinases (CDKs) emerge as an intriguing molecular subgroup. In human, this kinase family encompasses 21 members, involved in several key biological processes. Remarkably, 13 of these CDKs belong to the category of understudied kinases, and only 5 having undergone broad investigation to date. This knowledge gap underscores the pressing need to delve into the study of these kinases, starting with a comprehensive review of the less-explored ones.

Here, we will focus on the PCTAIRE subfamily of CDKs, which includes CDK16, CDK17, and CDK18, arguably among the most understudied CDKs members. To contextualize PCTAIREs within the spectrum of human pathophysiology, we conducted an exhaustive review of the existing literature and examined available databases. This approach resulted in an articulate depiction of these PCTAIREs, encompassing their expression patterns, 3D configurations, mechanisms of activation, and potential functions in normal tissues and in cancer.

We propose that this effort offers the possibility of identifying promising areas of future research that extend from basic research to potential clinical and therapeutic applications.

**Supplementary Information:**

The online version contains supplementary material available at 10.1186/s12943-024-02043-6.

## Introduction

Cyclin-Dependent Kinases (CDKs) constitute a family of proteins initially recognized as the principal regulators of the cell progression through the mitotic cell cycle [1]. However, confining the role of CDKs solely to cell cycle control is overly simplistic. CDKs play a pivotal role in DNA replication, repair, transcription, and numerous other critical processes by phosphorylating several different substrate proteins. Consequently, mutations in their coding genes disrupt normal cell physiology, contributing to a wide spectrum of pathologies, from cancer to neural disorders.

CDKs belong to the CMGC family of Serine/Threonine kinases, which also includes Mitogen-Activated Protein Kinases (MAPKs), Glycogen Synthase Kinase-3 beta (GSK3β), members of the Dual-specificity Tyrosine-Regulated Kinase (DYRK) family, and five additional genes encoding a more distant group of proteins known as CDK-like kinases (CDKL) [2]. Over the course of evolution, CDKs have undergone unexpected divergence and specialization, with their number increasing from 6 genes in the yeast genome to 21 genes in the human genome [3].

**Fig. 1:**
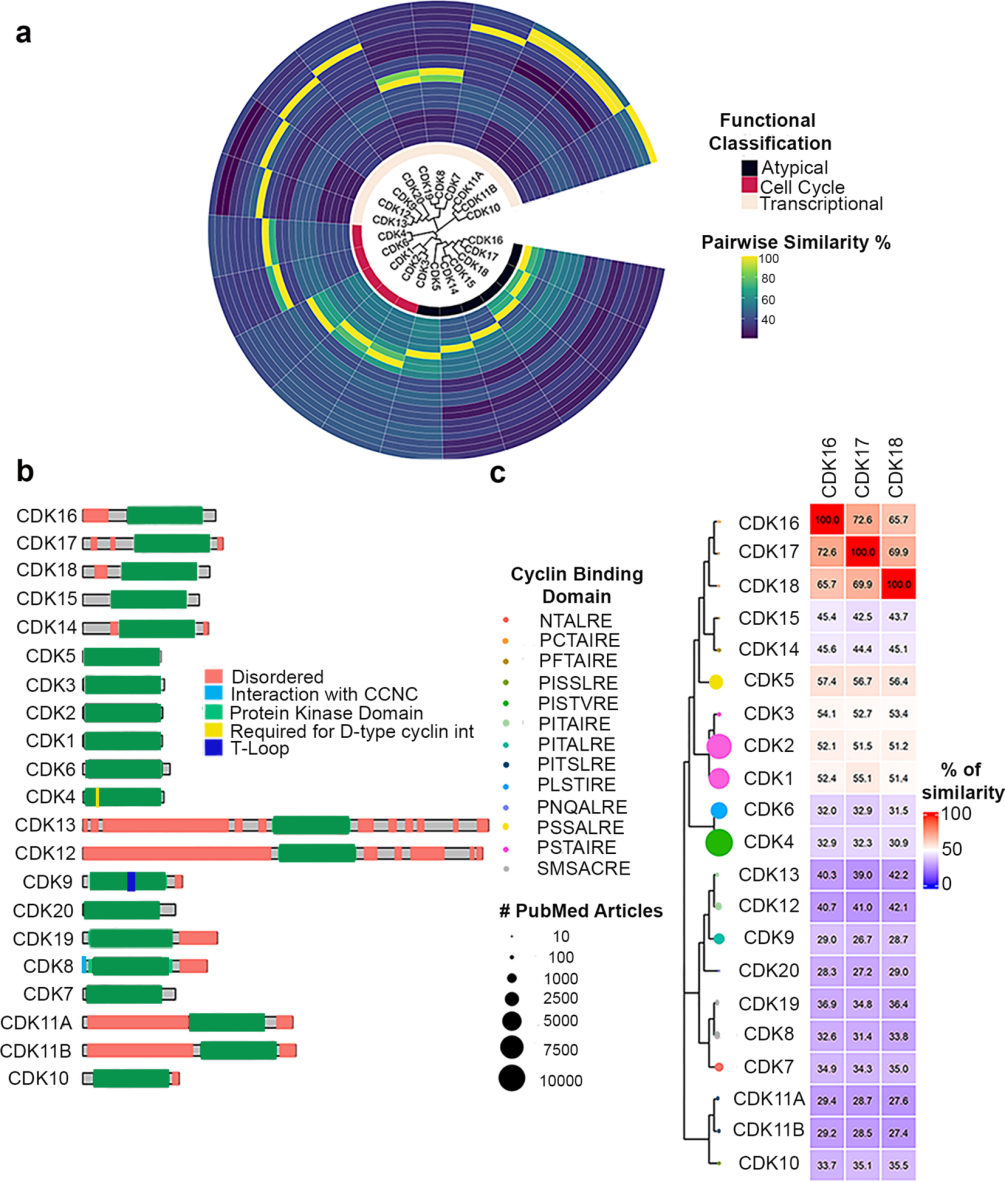
Sequence similarities of PCTAIREs with other CDKs. (**a**) Circular representation of human CDKs and associated annotations. The inner circle depicts functional classification of CDKs (cell cycle, transcriptional and atypical). All the remaining circles represent % of similarity among full length CDKs. Protein sequences were retrieved from Uniprot. Similarity scores were calculated with SeqinR [144] package and visualized with ggtree package [145]. (**b**) Schematic representation of CDKs generated using the drawProteins package [146]. Annotations were retrieved from UniProt database, last visited 30th November 2023 [147]. While protein kinase domain is well conserved among all CDKs, they diverge considerably in the length of N and C-terminal extensions. Colours are depicting UniProt annotation of the proteins. In the legend “Required for D-type cyclin interaction (int)” depicts a motif in CDK4 and CDK6 involved in their binding to cyclin D (CCND). (**c**) Phylogenetic tree of full length CDKs and associated annotations. Phylogenetic tree was constructed with MEGA X software [148], visualization/annotation was obtained with ggtree package [145] and Pubmed query was performed with rentrez package [149], last visited 30th November 2023. Node size indicates the number of articles where CDKs are mentioned. As shown, while CDK1, 2, 4 and 6 have all been mentioned more than 7000 times, PCTAIREs have been mentioned only a handful times. Different colours are depicting sub-family specific cyclin-binding domains. Similarity scores were calculated with SeqinR package [150] and plotted with complex heatmap package [151]

The 21 human CDKs are named from CDK1 to CDK20, with CDK11 encompassing two isoforms, encoded by two separate genes (CDK11A and CDK11B) [4]. These CDKs can be classified into three phylogenetic subgroups, based on their primary functions: (1) primarily involved in cell cycle regulation (CDK 1, 2, 3, 4, and 6); (2) primarily involved in transcription regulation (CDK 7, 8, 9, 10, 11, 12 and 13); and (3) atypical CDKs (CDK 5, and 14–20), each of which serves diverse, yet specific, functions (Fig. 1a) [3]. Among understudied and atypical kinases, there is the CDKL family, composed of five relatively underexplored human kinases: CDKL1, CDKL2, CDKL3, CDKL4, and CDKL5. This family has the highest sequence similarity to CDKs and CDKLs present a cyclin binding domain, although there is no evidence of interaction with cyclins. Little is known about CDKL1–4, their function and role(s) in human biology, whereas CDKL5 has been identified as a regulator of ciliogenesis [4–7].

The kinase domain characterizing CDK family spans about 250 amino acids and is the most conserved region among human CDK proteins, with homology ranging from 40 to 65.5%. Within the kinase domain, the HRD and DFG motifs are highly conserved and essential for kinase activation. As their family name implies, CDKs require an activating partner, classically cyclins, to function properly [8]. However, also phosphorylation by CDK-activating kinases (CAKs) on specific threonine/serine residues (*e.g.* Thr161 in CDK1 or Thr160 in CDK2) is required for the activation of some cycle-related CDKs (such as CDK1, CDK2, CDK4 and CDK6) and transcriptional CDKs (CDK9, CDK12 and CDK13). In vertebrates, the active CAK complex is composed of three subunits: CDK7, its cyclin partner Cyclin H and the RING finger protein MAT1. This complex is necessary to phosphorylate a threonine residue (e.g. Thr161 in CDK1) in the T-loop, which is a critical step to obtain full CDK activation [9–12]. These residues are likewise faithfully conserved across human CDKs. Similarly, tyrosine residues at the N-terminus of the kinase domain (*e.g.* Tyr15 in CDK1), acting as inhibitory phosphorylation sites, are also highly conserved.

CDKs can also be classified based on the sequence of their cyclin binding region (Fig. 1b-c). For instance, CDKs can be divided into PSTAIREs (comprising CDK1-3), PSSALRE (CDK5), PFTAIREs (CDK14-15), PCTAIREs (CDK16-18) and PI(L)STV(I)REs (CDK4-6) (Fig. 1b, c). It is important to note that the last two residues of the cyclin-binding region (XXXXXRE, where X represents any amino acid) are also highly conserved. Looking at the phylogenetic trees (Fig. 1a, c), as also reported by others [3], CDK4 and CDK6 (PI(L)STV(I)RE) equally and very early diverge from CDK1-3 (PSTAIRE) that are therefore phylogenetically more similar and better cluster to CDK5 subfamily (*i.e.*, PSSALRE, PFTAIRE and PCTAIRE).

Looking at literature data, it is quite evident that while cell cycle-related CDKs have been extensively studied, most of the other CDKs are far less explored (Fig. 1c, see dot size), suggesting that the study of their roles in human pathophysiology might still hold some surprise. Accordingly, many atypical and transcriptional kinases (ranging from CDK10 to CDK20) are part of the Dark Kinase database (DKK; https://darkkinome.org), [13] a consortium focused on gaining a better understanding of the approximately 160 kinases whose function in human biology remains poorly understood. DKK aims to provide data and resources for these understudied kinases to the research community, reinforcing the importance of elucidating the role and potential therapeutic significance of understudied kinases in human diseases.

In line with this idea, we have reviewed the available information on one subfamily of these under-researched CDKs, the PCTAIREs (CDK16, 17, and 18), offering new insights into their structure and function and aiming at a better characterization of their still uncertain role in both cell biology and cancer.

## Sequence homology comparison underscores the uniqueness of PCTAIREs

PCTAIREs, firstly cloned in 1992 [14, 15], consist of approximately 500 amino acids in length. In a phylogenetic analysis, PCTAIREs are shown to exhibit substantial similarity to the other CDKs, notably 42–46% with PFTAIREs (CDK14 and 15), 58% with CDK5 and 52–54% with canonical CDKs (CDK1, 2, and 3), as illustrated in Fig. 1c. It is well-established that cyclin binding triggers structural reconfiguration in CDKs, enabling them to carry out their kinase activity [16–18]. Mutagenesis experiments and structural studies have shown that pan-kinase conserved motifs, like HRD and DFG, are indispensable for kinase activation [19–21]. As depicted in the multiple sequence alignment (MSA), HRD and DFG motifs, along with their encompassing residues, are highly conserved among all CDKs (Supplementary Fig. 1).

Furthermore, canonical CDKs require phosphorylation by CDK-activating kinases at specific threonine residues (*e.g.* CDK1 at Thr161, CDK2 at Thr160) to transition from an inactive to a fully active state. Notably, in P(C/F)TAIREs and CDK5, the corresponding residue is a serine, indicating the likelihood of similar activation mechanisms governing the full activation of these CDKs (Supplementary Fig. 1). This possibility is further supported by observations that the substitution of threonine to serine in position 160 of CDK2 does not diminish its kinase activity but does impact on its fine-tuning during cell cycle progression [22]. This finding could represent a significant distinction in the regulation of kinase activity between canonical and non-canonical CDKs. Despite high similarity in their kinase domains (Supplementary Fig. 2), all PCTAIRE and PFTAIRE proteins diverge quite significantly from each other in their N-terminal regions (Fig. 1b and Supplementary Fig. 1). Notwithstanding the low similarity in N-terminus, a well-conserved Protein Kinase A (PKA) substrate motif (R-R-X-S) is detected in PCTAIREs (Supplementary Fig. 1), suggesting the existence of common post-translational modifications among PCTAIREs. Indeed, several evidences have shown that N-termini phosphorylation of PCTAIREs are important to fine-tune their activity/function [23–28].

## PCTAIREs 3D structure analysis

Crystallographic studies and the development of three-dimensional (3D) structures have proven to be indispensable not only to gain a comprehensive understanding of the kinase function and its possible interactions, but also for the design of specific pharmacological inhibitors [29–31]. However, crystal structure data for PCTAIREs remain scarce. Currently, only the structure of CDK16 has experimentally been studied [32]. Sarah et al. meticulously studied the crystal structure of the CDK16 kinase domain, revealing its adherence to the classical bi-lobal architecture, interspersed with short insertions that contribute to the characteristic folding of CDK family [32]. This investigation also illuminated the structural compatibility of CDK16 kinase domain with the cyclin Y/14-3-3 complex. This interaction is believed to be important for CDK16 kinase activity, and it may be mutually exclusive with the binding of specific CDK16 inhibitors, such as Dabrafenib and Rebastinib [32]. Noteworthy, peculiarities within the CDK16 structure include a partially inverted DFG motif and distinctive conformations of the C-terminal extension and CDK/MAPK motif, shared between CDKs and MAP kinases. This motif forms an additional protein-protein interaction surface, unique to the CDK family. For instance, in CDK2 the CDK/MAPK domain facilitates its interactions with the CDK-binding protein CKS1. It has been proposed that CKS1, by binding to CDK2 in conjunction with the CDK inhibitor p27Kip1, enables the ubiquitination and subsequent degradation of p27Kip1, thus indirectly regulating CDK2 activity [33, 34]. Since the CDK/MAPK motif is involved in CDKs activity regulation, the observed structural differences likely imply that the surfaces of CDK2 and CDK16 have evolved to mediate distinct protein-protein interactions, consequently serving different biological functions [32].

While the existing structural data support the notion that CDK16 (and potentially other PCTAIRE kinases) can interact with cyclins [32], other experimental evidence suggests that such interaction alone might be insufficient to trigger full PCTAIRE kinase activity. As elegantly demonstrated by Shetate et al., bacterially expressed cyclin Y (CCNY) by itself does not activate CDK16 kinase activity [35]. This observation finds partial support in work from Hernandez et al., showing that a purified CDK16-CCNY complex exhibits only weak ability to phosphorylate MBP (Myelin Basic Protein) protein [36]. Therefore, unraveling if and how the 3D conformation of PCTAIRE kinases changes upon cyclin binding and transitions between active and inactive state, much like what has been already established for cell cycle CDKs, is of paramount importance.

Given the absence of a direct comparative analysis, elucidating the configurational differences between active and inactive state in CDK16 (and other PCTAIRE kinases), we undertook a comparative approach by examining the CDK16 structure in relation to other CDKs. To this aim we used the structure of those CDKs that have the highest similarity with PCTAIREs in their kinase domains and for which both inactive and active 3D conformations are available. To achieve this, we utilized the available structures of CDK5, considered the human homolog of the yeast Pho85 kinase and a prototype of the PCTAIRE and PFTAIRE family, which shares 57% sequence homology with CDK16, as well as the structures of CDK1 and CDK2, both of which exhibit 52% sequence homology with CDK16 (Fig. 1c) [3].

**Fig. 2:**
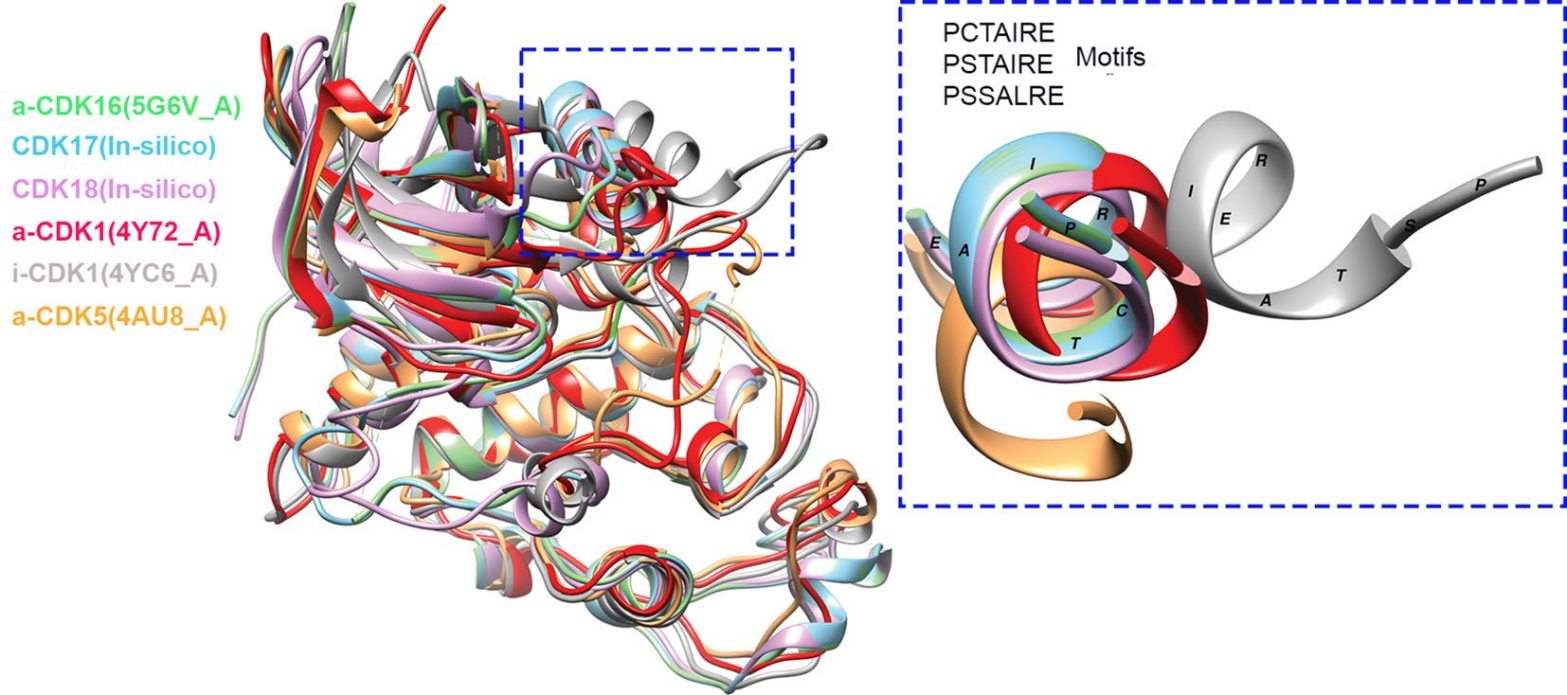
Structural comparison between PCTAIREs and canonical CDKs. CDK1, 5 and 16 structures were retrieved from PDB database. [PDB]-ID: CDK1[4Y72_A], CDK1[4YC6_A], CDK5[4AUA8_A], CDK16[5G6V_A]. CDK17 and CDK18 structures were generated by homology-modelling using SWISS-MODEL [152]. The italic letter preceding protein name indicates: *a_ (active) and i_ (inactive)* configuration of the structure. For full description see Supplementary Information. All structures were superimposed onto CDK16 structure. Blue dot-line rectangle depicts the cyclin binding domains. As shown cyclin binding domain of inactive CDK1 (PSTAIRE) has different 3D coordinates compared to the other structures (active CDK1 (PSTAIRE) and active CDK5 (PSSALRE), inactive CDK16 and in silico 3D structures, based on homology modelling, of CDK17 and 18 (PCTAIRE)). On the right, zoom-in view of the same cyclin-binding regions. Notably, all PCTAIREs (CDK16 in inactive state) cyclin-binding motifs have much closer trajectory to the respective motif of active CDK1 (PSTAIRE), compared to active CDK5 cyclin-biding motif (PSSALRE). Visualization was obtained with the Chimera software [153]

In the supplementary information, we present a comprehensive description of our 3D structure assignment, distinguishing between predicted active and inactive conformations, alignment, and superimposition, related to structural comparison (Supplementary Figs. 3, 4, 5, 6, 7). This analysis has yielded several interesting findings. Firstly, CDK16 shares a slightly higher overall structural similarity with active structures of CDK1, 2 and 5, compared to inactive ones (Supplementary Fig. 3b, c). Then, specifically, the PCTAIRE domain of CDK16 aligns better with the active PSTAIRE domain of CDK1, compared to other structures (CDK1 inactive; CDK2,5 either active/inactive) (Supplementary Fig. 4c, d inset 1, Supplementary Fig. 5 inset 1, Supplementary Fig. 6 inset 1). Lastly, overlapping geometries in the cyclin binding domains of PCTAIREs (especially between CDK16 and CDK17) were also observed (Fig. 2 and Supplementary Fig. 7).

Collectively, these structural analyses suggest that PCTAIREs (especially, CDK16 and 17), may be able to adopt an active conformation even in the absence of a specific cyclin or a regulatory binding protein. These observations also underscore the importance of examining the structure of a protein as a whole, not limiting the study to the catalytic domain, for a comprehensive understanding of the kinase activity regulation. In support of that, several studies have shown that PCTAIREs are phosphorylated at their N-terminus [23–25, 27, 28, 26–38], as will be discussed in detail later in the PCTAIREs interactions and functions paragraph.

Our in-silico findings also stimulate the formulation of new hypotheses and raise key questions, especially regarding the molecular mechanisms governing the CDK activity and the extent to which these structural variances differentiate PCTAIREs from canonical CDKs. Comparative crystallographic studies (active vs. inactive) states of PCTAIREs are likely to provide a clearer and more definitive picture of these aspects and may pave a way to design novel chemical inhibitors for these proteins.

The role of Cyclin binding to PCTAIREs and its potential impact on the activation of these kinases remains far from being a complete picture. Although some evidences showed that cyclin Y (CCNY) and cyclin YL1 (CCNYL1) could bind CDK16 and, possibly, regulate its kinase activity, it is still unclear whether this binding is stable, direct and/or whether it requires the binding of 14-3-3 protein to a previously phosphorylated CDK16. The binding with 14-3-3 proteins potentially increases CDK16 basal kinase activity [27, 35, 39, 40]. Accordingly, the binding between CCNY or CCNYL1 and CDK16 endogenous proteins is generally challenging to detect [27, 35, 40]. The data regarding the possible reported binding of CDK17 with CCNY, Cables, or cyclin A2 are equally uncertain [24, 41]. Conversely, a cytoplasmic interaction between cyclin A2 (CCNA2) and CDK18 has been observed at the endogenous level and this binding is likely important for CDK18 activation [24]. Collectively, these findings suggest that CDK16 and CDK17, unlike cell cycle CDKs, might also be activated through still not well defined non-canonical pathways.

Notably, size exclusion chromatography and gel filtration demonstrated that monomeric PCTAIRE-1 (CDK16) was active as a protein kinase. The activity of CDK16 detected in this context was comparable to the one of CDK5 when associated with its activating binding partner [23]. These findings lend support to the notion that CDK16 could possess kinase activity independently from the binding to a regulatory subunit. However, this experimental approach could not exclude the possibility that CDK16 interacts with a small protein not exactly distinguishable by size exclusion chromatography and/or that it may exhibit significantly enhanced protein kinase activity upon binding to the proper regulatory subunit.

## PCTAIREs inhibitors

Although activation mechanisms of CDK16 and CDK17 remain a subject of ongoing exploration, it is clear that all PCTAIREs possess inherent kinase activity [24, 42, 43]. The development of specific inhibitors for this CDK family could, therefore, greatly contribute to our understanding of their biological functions and potentially offer new opportunities of clinical application. Yet, our current knowledge in this area is still very limited and requires further research efforts.

Significant strides in kinase structural biology have greatly enhanced our comprehension of various aspects of kinase biology and expedited drug discovery efforts [44]. The structural organization of the catalytic domain in all eukaryotic protein kinases reveals a common bi-lobal fold, featuring a smaller N-terminal lobe and a larger C-terminal lobe, connected by a “hinge.” ATP binds to a highly conserved pocket situated deep between the two lobes and forms hydrogen bonds within the “hinge” region [44].

Building upon this foundational knowledge, Vijayan et al. conducted a study that categorized kinases into two groups, based on their DFG-out conformational status and inhibitor selectivity. Group 1 includes kinases with classical DFG-out conformation, possessing a large allosteric pocket that can be targeted by both type I inhibitors (binding in the DFG-in conformation, active kinase state) and type II inhibitors (binding in the DFG-out configuration, inactive kinase state) [45]. Group 2, on the other hand, encompasses kinases with non-classical DFG-out configurations, which have a smaller allosteric pocket when activated, and can be targeted solely by type II inhibitors [19]. Experimental evidence shows that CDK16 can be inhibited by both type I and II inhibitors, such as Dabrafenib and Rebastinib, respectively [32].

There are not many studies that tried to identify specific PCTAIREs inhibitors. Research aimed at identifying a specific and selective CDK14 inhibitor led to the production of the compound FM-04-159-2, also referred to as Pan-TAIRE (CDK14-18), that potently and reversibly inhibit all PCTAIREs [46]. Recent advancements have led to the development of optimized N-(1 H-pyrazol-3-yl)pyrimidin-4-amine moieties that target PCTAIREs with high potency in the nanomolar range (20–120 nM for PCTAIREs and 80–150 nM for PFTAIREs) [47].

An alternative and potentially expedited approach to identify kinase inhibitors could be represented by drug repurposing studies. In that regard, pan-kinome study using 243 clinically approved or investigational kinase inhibitors to identify novel targets by Klaeger et al. offers an excellent resource [48]. Their mass spectrometry-based chemical approach, known as “Kinobead”, measured the affinity of each inhibitor towards different kinases [49]. Notably, their data revealed that most kinase inhibitors, including CDK inhibitors, have several additional targets beyond their designated primary ones.

**Fig. 3:**
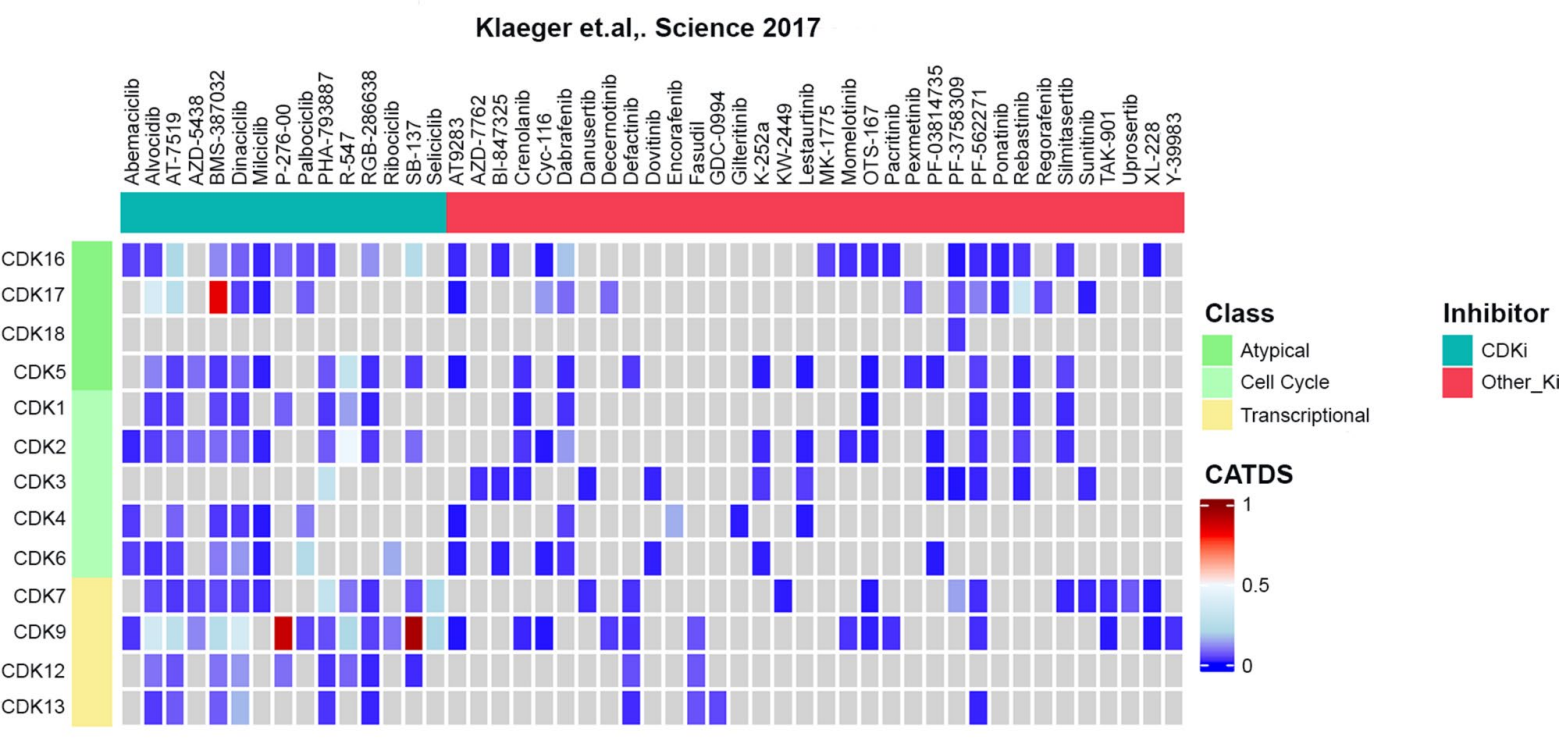
Efficacy of kinase inhibitors against PCTAIREs. Heatmap representation of selectivity score, termed CATDS (Concentration And Target Dependent Selectivity), between CDKs and small molecules, includes CDK inhibitors (CDKi) and other kinase inhibitors (Other_Ki). Data were retrieved from [48] and visualized using complex heatmap package [151]

Exploring data from Kinobead [48] for CDKs/PCTAIREs, we observed that most CDK inhibitors (CDKi) are pan-CDKi, including CDK16 and CDK17 as targets, and exhibit low “Concentration And Target Dependent Selectivity (CATDS)” scores (Fig. 3). Only three compounds show a higher degree of selectivity: BMS-38,702_(CADTS=0.84)_ for CDK17 and P-276-00_(CADTS=0.89)_ and SB-1317_(CADTS=0.95)_ for CDK9. Interestingly, while CDK9 represents the designated target for both P-276-00 and SB-1317 [50–55], why and how BMS-387,032 is highly selective towards CDK17 is still unclear. Further studies and crystallography analysis of CDK17-BMS-387,032 could provide valuable insights into how BMS-387,032 can be repurposed to selectively target CDK17. Of note, using the same approach (Kinobead), an independent group also showed that BMS-387,032 is highly potent towards CDK17 (CDK17_IC50_ = 90nM vs. designated targets (CDK9_IC50_ = 50nM, CDK7_IC50_ = 170nM and CDK2_IC50_ = 300nM) in primary Chronic Lymphocytic Leukemia (CCL) cell lines [56]. Finally, it has been recently proposed that certain CDK inhibitors could have strong affinity for PCTAIRE-family CDKs, particularly the CDKi-73 for CDK16 and CDK17 [57].

In the case of CDK18, a unique scenario emerges. As easily observed from the heatmap reported in Fig. 3, while most CDKs could be potentially inhibited by several different compounds, CDK18 exhibits limited engagement with only one compound, PF-3,758,309 (a PAK4 inhibitor), demonstrating low CATDS score for CDK18. This observation will likely merit further studies aiming at understanding why CDK18 behaves so differently from all other CDKs in term of kinase activity inhibition. By studying the ATP binding domain of CDK18 in comparison to the ones of CDK16, CDK1 and CDK2, we did not observe significant differences, suggesting that basal 3D CDK18 protein structure is not the principal cause of the differences in small molecule binding.

To advance our understanding and facilitate chemical and pharmacological research, it is crucial to obtain experimental 3D structures for CDK17 and CDK18. Such tools will be instrumental to the development of protein-specific inhibitors, aiding in the exploration of both their unique and shared functions. The availability of X-ray crystal structures would also be fundamental for computer-aided drug design (CADD) of new peptide inhibitors, with enhanced specificity. Indeed, ATP-binding domain inhibitors, which are the most common CDK competitive inhibitors, do not target only one CDK: for example, R-Roscovitine inhibits CDK1, CDK2, CDK5, CDK7, and CDK9 [58]. A deeper understanding of the structural differences between various CDKs’ ATP binding sites, which reveal crucial ligand-receptor interactions specific to each CDK, would significantly enhance the specificity of new small molecules or peptides [59]. These molecules could act as reversible ATP competitors or allosteric inhibitors. Additionally, since CDK activation and regulation primarily depend on protein-protein interactions (PPIs), other strategies, informed by 3D modeling, focus on designing new peptide inhibitors that interfere with PPIs [58, 60, 61]. For example, the cyclin recognition motif in the CDK-inhibitory protein p27Kip1 has been used to design and synthesize a series of cyclic peptides that disrupt the CDK-cyclin interaction. Furthermore, predicting the interaction geometry between proteins and peptides could be very useful to design and screen potential peptides capable of binding the active site of a specific CDK, thereby inhibiting its catalytic activity [62]. The same is true for the design of a new class of molecules able to specifically degrade target proteins, like PROTAC [63]. Alternatively, artificial intelligence-driven tertiary structure prediction models could be developed to support the proper design of drugs that target these understudied kinases [64].

### PCTAIRE expression in normal human tissues

Since the beginning, studies on PCTAIRE presented compelling findings that these kinases display distinctive expression patterns across diverse tissues. In general, they were reported to be prominently expressed in differentiated tissues, including brain, testis, post-mitotic neurons, and elongated spermatids, with some differences among the three different CDKs. Specifically, CDK16 is more abundant in differentiated tissue but displays a relatively ubiquitous expression profile. Conversely, CDK17 and CDK18 exhibited more limited patterns of expression, primarily observed in the brain, kidney, testis, and intestine [14, 15, 42]. An overview of PCTAIREs expression in different human tissues (retrieved from Genotype-Tissue Expression (GTEx) Portal) is reported in Supplementary Fig. 8.

Being subsequent experimental analyses scarce, we further explored publicly available databases, such as https://www.proteinatlas.org/, to corroborate these initial findings. These analyses confirmed that CDK16 exhibits a lower degree of tissue specificity, with the highest expression levels noted in skeletal muscle and brain. CDK17, on the other hand, appears to be most abundantly expressed in the brain, lymphoid tissues and reproductive organs, in both males and females. In contrast, CDK18 expression appears to be constrained primarily to the brain, notably in the spinal cord, and enriched in muscular tissues, especially in the heart.

Complementing these observations, single-cell RNA expression data further confirmed the distinctive expression profiles of these kinases. CDK18 exhibits high expression in oligodendrocytes, while CDK17 is prominent in glial, neuronal, lymphoid, and testicular (germinal) cells. CDK16, conversely, presents a widespread expression pattern across different cell types, with a peak in well differentiated spermatids.

### *Pctaire* expression and functions in murine tissues

In-depth analysis and comparison of available databases that annotate RNA and/or protein expression of murine *Pctaire* genes, such as those accessible through https://www.ebi.ac.uk/ebisearch/about or https://www.informatics.jax.org/, have further confirmed a similar expression pattern in mice. In line with previous observations [27, 65], *Cdk16, Cdk17 and Cdk18* are predominantly expressed in brain, skeletal muscles, heart and to varying degrees in other organs/tissues. It is worth noting that while both *Cdk16* and *Cdk17* are expressed during embryonic development, at least from E11 to E18, there is no data reporting the expression of *Cdk18* during mouse embryogenesis.

The expression patterns of Pctaire kinases in mice well align with the phenotypes observed in correspondent knockout models. Mikolcevic et al. generated a conditional knockout (KO) model for *Cdk16*, revealing that Cdk16KO mice were born at expected frequencies and did not exhibit any obvious phenotypic differences from control littermates. However, homozygous Cdk16KO male mice displayed sterility, characterized by impaired sperm motility, dyskinesia, and various morphological abnormalities in sperm, including malformed heads, cytoplasmic excess, and structural defects in the annulus region [27].

Also *Cdk17* and *Cdk18* KO mice have been generated, but the results are as yet unpublished [66]. However, detailed information can be found on the International Mouse Phenotyping Consortium (IMPC) website (https://www.mousephenotype.org/). In the case of Cdk18KO mice, the use of CRISPR-CAS9 technology in the C57BL/6NJ strain led to an intragenic deletion, resulting in early protein truncation. Notably, these mice did not display any distinctive phenotypic characteristics, except for a notable increase in circulating creatinine levels, suggesting possible renal dysfunction that warrants further investigation (https://www.mousephenotype.org/data/genes/MGI:97518).

Cdk17KO mice were also generated with the CRISPR-CAS9 technology resulting in the deletion of *Cdk17* Exon 4 in the C57BL/6NJ strain, that produced a protein truncated after residue 94. These mice exhibit several intriguing features. Using the Open Field test, a test commonly used to assess anxiety and exploratory behaviors, it was reported that heterozygous Cdk17WT/KO female mice were significantly hyperactive, manifesting increased movement between locations compared to control. Homozygous Cdk17KO male mice showed elevated vertical activity, indicating a higher propensity for jumping or rearing in the same test. It is worth noting that knockout of *Cdk17* gene in mice led to partial preweaning lethality, particularly evident in female embryos. In particular, percent of homozygous KO mice at birth dropped from the expected 25% to only 7.8% (7 out of 90). Among the 7 born pups, only one was female, emphasizing the prominent phenotype in females as opposed to males. A gross morphology embryo E18.5 phenotypic assay revealed that 28.6% of analyzed female embryos displayed abnormalities (https://www.mousephenotype.org/data/genes/MGI:97517). These findings suggest that Cdk17 expression plays a pivotal role in the complete development of mouse embryos, particularly in females, indicating possible gender-specific roles.

Early attempts to generate constitutive knockout of Cdk16 failed, as male murine ES cells lacking Pctaire-1 did not demonstrate germline transmission [23]. This observation suggests that Cdk16 may be required for the formation of viable sperm or could be necessary during embryogenesis, as partially observed for Cdk17. The former hypothesis appears more likely and is supported by the generation of Cdk16 conditional KO models [27]. Furthermore, cyclin Y-like 1 (Ccnyl1) KO mice, which develop normally but exhibit male infertility associated with asthenozoospermia (reduced sperm motility) also supports this hypothesis, as Ccnyl1 KO led to a substantial reduction in Cdk16 expression in the testis, suggesting its involvement in regulating Cdk16 protein stability. Nevertheless, it remains to be determined whether Ccnyl1 contributes to the regulation of endogenous Cdk16 kinase activity [40].

In summary, in vivo studies in mice provide insights into the vital roles of Cdk16 and Cdk17 in distinct developmental phases and organ physiology. These findings offer intriguing prospects for further investigations into the underlying molecular mechanisms. The exploration of gender-specific effects observed in Cdk17KO mice during embryonic development and neuronal activity, is particularly relevant and could yield valuable information regarding protein-protein interactions and PCTAIRE kinases unique functions.

## PCTAIRE interactions and functions

As previously mentioned, PCTAIRE kinases remain a relatively understudied group of kinases, and consequently, only limited information concerning their molecular roles and interactions is currently available. Given that PCTAIREs exhibit high expression in specialized tissues such as brain, testis, post-mitotic neurons, and elongated spermatids, much of the research has focused on investigating their potential contributions to cellular differentiation and specific biological contexts [65]. To enhance clarity, we will review the existing information in dedicated subheadings for each CDK.

### CDK16

The initial indications about CDK16 interaction with potential regulatory subunits emerged from early observations indicating that CDK16 kinase activity was lost when immunoprecipitated from the testis in a high saline buffer, suggesting that the dissociation of regulatory factors or protein denaturation could compromise its activity [65]. Additionally, bacterially expressed CDK16 was able to induce MBP phosphorylation when mixed with mammalian cell lysates, indicating a potential requirement for specific post-transcriptional modifications or activators for its kinase activity [65]. Two-hybrid and immunoprecipitation assays found p11 or 14-3-3 as CDK16 interacting partners in the brain [65, 67]. Nevertheless, the functional implications of these interactions remain to be fully elucidated. Furthermore, it was shown that CDK5/p35 complex by phosphorylating CDK16 at serine 95 (Ser95) increases its kinase activity [28]. Through forward genetic analysis in C. elegans, cyclin Y (CYY-1) was identified as a binding partner for CDK16. This interaction was found to be necessary for targeting presynaptic components to the axon, providing an initial indication of CCNY potential role as a CDK16-activating regulatory subunit, at least in overexpressing models [68]. Subsequent studies further confirmed the binding of CCNY to CDK16 through two-hybrid screening and overexpressing cells, emphasizing the importance of this binding for CDK16 kinase activity [27]. Notably, CCNY binding to CDK16 occurred at the plasma membrane and required a region upstream of the kinase domain. This binding was found to be inhibited by the phosphorylation of Ser153, a potential PKA phosphorylation site, making CDK16 the first CDK in which cyclin binding was shown to be phosphorylation-dependent [27]. Indeed, it was shown that PKA phosphorylates CDK16 in four different serine residues, being Ser153 the main phosphorylation site [23]. Another study showed the interaction between CDK16 and CCNYL1 and delineated the role of this complex in regulating spermatogenesis [40]. A recent study showed that AMPK phosphorylates CCNY at Ser326 to further promote CCNY-CDK16 interaction and increase its kinase activity [69]. It is worth noting that, in both brain and testis, immune-depletion of CCNY does not affects the expression of CDK16 in protein lysates (and vice versa). These data suggests that either only a small portion of CDK16 is bound to CCNY or that this binding is particularly weak and subject to high variation depending on the experimental conditions used [39].

Despite our limited understanding of how CDK16 becomes fully active, several convincing experiments have demonstrated its role in regulating the activity of differentiated neurons and spermatozoa [23, 27, 36, 40, 43, 68, 70–73].

A handful study also link CDK16 to various type of neuropsychological disorders, such as learning deficiency, altered social behavior in experimental models and Alzheimer diseases [74–77]. A quite recent and intriguing observation links CDK16 to the regulation of autophagy [69]. In this regard, since CDK16 functions in vesicular transport and in actin cytoskeleton organization are both relevant for autophagy, it is proposed that CDK16 could be involved in bringing autophagosomes in close proximity to lysosomes or in balancing the cellular decisions between autophagy and apoptosis [78]. As such, CDK16, by inducing autophagy, could be associated with many different diseases, including inflammatory, neurodegenerative and tumorigenic ones [78]. Finally, recent evidences report that CDK16 may also regulate myogenic differentiation, as its overexpression increases the expression of known myogenesis markers, Myosin Light Chain (MHC) and Troponin C, whereas its silencing elicits the opposite effect [79].

### CDK17

Even less information is available in literature regarding the physiological and pathological roles of CDK17.

CDK17 expression is predominantly cytoplasmic/membranous in normal human tissues, though in cultured cells it may also translocate to the nucleus. CDK17 is highly expressed in terminally differentiated neurons, particularly those found in hippocampal regions and olfactory bulbs [42]. Its expression is significantly upregulated in patients with Alzheimer’s disease and Mild Cognitive Impairment [38, 77]. Interestingly, Genome-Wide Association Study (GWAS) has linked CDK17 to the glycerophospholipid metabolism pathway [80]. CDK17 expression is strongly correlated with and significantly inhibits viral infections, in experimental models [81].

Like CDK16, CDK17 kinase activity appears to diminish when it is immunoprecipitated from rat brains in high saline conditions [42]. While CDK17 has been shown to interact with TRAP (Tudor repeat associated with PCTK2) and Cables1 (Cdk5 and Abl enzyme substrate 1), neither of these interactors could stimulate CDK17 kinase activity in in vitro [41, 82].

Notably, a study employing a library of 354 human kinases and kinase-related open reading frames that are activated through membrane recruitment by adding a myristylation signal, identified CDK17, along with CDK18, as potential inhibitors of the autophagic process [83].

While the research did not delve deeply into the role of these two PCTAIRE kinases in autophagy, it strongly supports the notion that PCTAIRE kinases in general, and CDK17, in particular, could be involved in the regulation of vesicle internalization and trafficking in various physiological and pathological contexts, particularly at the plasma membrane. Nonetheless, a more extensive body of experimental research will be essential to fully unravel its roles in physiological and pathological processes.

### CDK18

In mature tissues and cell lines, CDK18 predominantly localizes in the cytoplasm and at the plasma membrane [73, 74]. In accordance with its expression in adult tissues, the majority of information regarding CDK18 functions pertains to studies in the field of neurology. A proteomic investigation revealed that treatment with antidepressants in rats led to an upregulation of CDK18 in the hippocampal regions [84]. Furthermore, transcriptome analyses have shown that CDK18 is upregulated in the central nucleus of the amygdala following chronic alcohol consumption [85]. In the context of remyelination, CDK18 plays a role in oligodendrocyte precursor cells by directly promoting their differentiation through the activation of the mitogen-activated protein kinase ERK pathway [86]. Interestingly, like CDK16 and 17, altered expression, phosphorylation of CDK18 has been reported in AD cell line, animal model and patient samples [38, 77, 87].

CDK18 was shown to interact with cyclin A2 and displayed kinase activity, which is further controlled by PKA through Ser12 phosphorylation [24]. CDK18 was shown to undergo vasopressin induced phosphorylation [37, 88, 89], and is involved in plasma membrane localization of aquaporin-channel (AQP2) [90]. Perturbations in the vasopressin-AQP2 signaling pathway have been associated with nephrogenic diabetes insipidus (NDI) [91]. The observation that CDK18KO mice display altered circulating creatinine levels, possibly indicative of renal dysfunction, somehow supports the link between CDK18 expression and diabetes onset and development, a hypothesis that might merit future investigation. CDK18 has been also observed to have slightly higher expression in diabetic pancreatic islets vs. normal islets [92], and genome-wide association studies have hinted at a possible link between CDK18 polymorphism and type 2 diabetes, particularly in African Americans [93].

Collectively, mounting evidences suggest that PCTAIREs have diverse functions in differentiated adult cells, particularly in neuronal ones, in which all three of these proteins have been implicated in controlling neurite outgrowth and vesicular trafficking. It is also noteworthy that PCTAIREs seem to play roles in maintaining the physiology of organs in the genitourinary system, such as the kidneys and testes, which typically develop from the intermediate mesoderm. However, new, more sensitive, and specific methods for identifying and studying protein-protein interactions [94, 95] are needed to provide further insights into the interactome of PCTAIRE kinases, shedding light on how they become fully active and regulate cellular physiology. With the assistance of high-throughput technologies and the development of more relevant in vitro and in vivo models, we anticipate that a deeper understanding of their roles and potential interactions, both during embryonic development and in the pathophysiology of adult organs, will be achieved in the next future. Supplementary Table 1 summarizes the most solid observations available on PCTAIRE functions, known substrates and interactors, providing an immediate overview on their possible roles.

## PCTAIREs and cancer

In spite of the general dearth of knowledge surrounding the PCTAIRE protein family, it can be said that all information gathered so far point to roles in signal transduction pathways, altogether potentially linked to the onset and progression of human cancer. Although this topic has been explored in only a limited number of studies, the emerging evidence supports the potential participation of PCTAIREs in the regulation of several critical hallmarks of cancer, including cell proliferation, DNA repair, and apoptosis. These burgeoning insights are poised to serve as the foundation for forthcoming investigations, which may then pave the way to innovative therapeutic strategies. In the following section, we present a concise overview of the expression and mutation patterns of PCTAIRE proteins in human cancer, along with the available data on the specific roles of each family member in different cancer types.

### PCTAIRE expression and mutation in human cancers

**Fig. 4:**
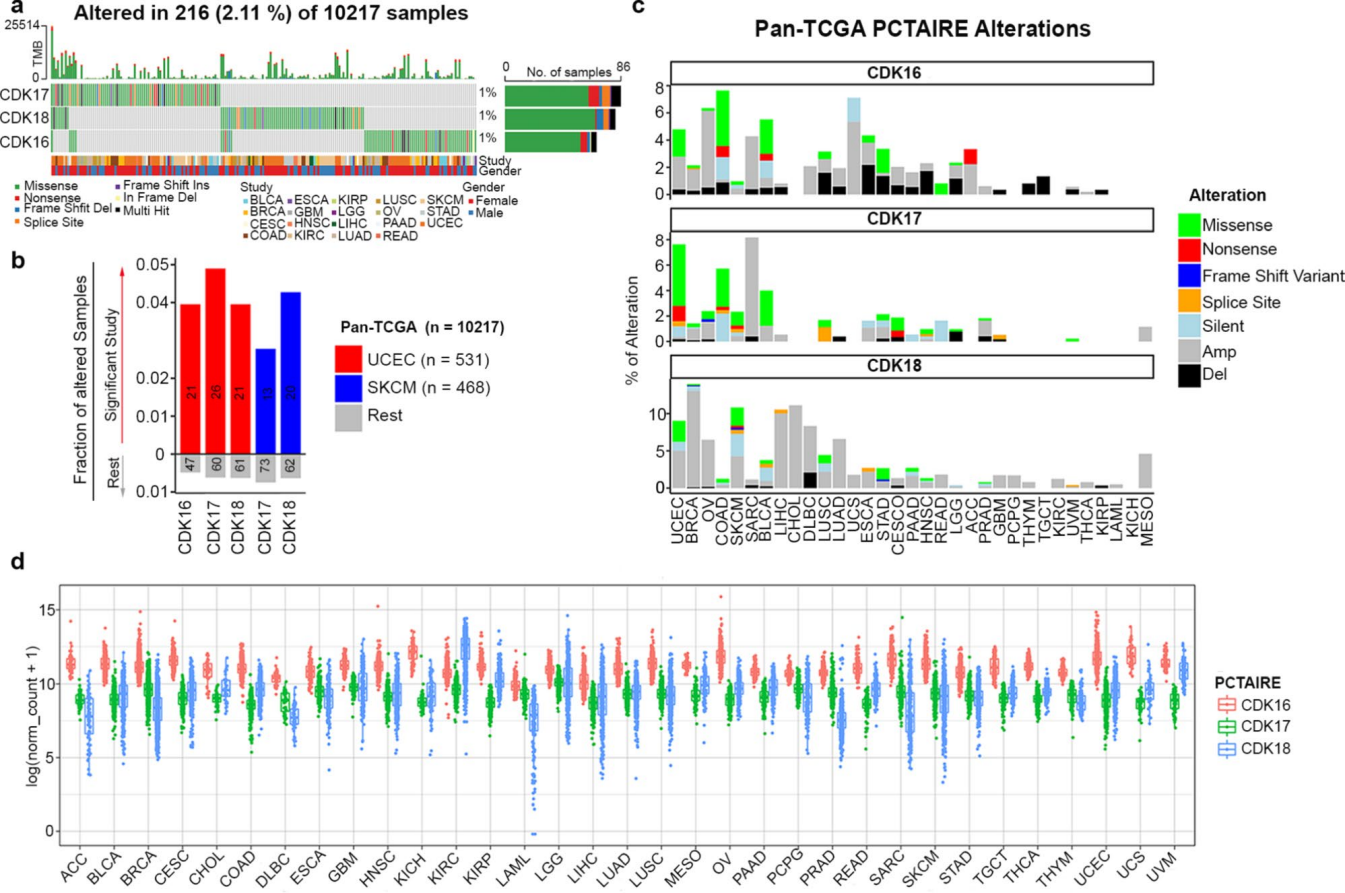
PCTAIRE mutation/expression analysis in Pan-TCGA datasets. (**a**) Oncoplot displaying PCTAIREs mutations in Pan-TCGA samples. Mutation and clinical data for all 33 TCGA studies were downloaded from GDC database [154], processed and visualized using Maftools, evaluating how many patients resulted mutated in PCTAIREs [155]. To simplify the visualization, only the 19 studies (out of 33), reporting PCTAIRE mutations are shown in the panel. TMB reports the levels of mutation observed (mutation/megabases). Color codes identify the TCGA studies evaluated and the mutation type identified for each PCTAIRE subfamily members (missense, nonsense etc.). (**b**) Graph displaying TCGA studies in which PCTAIREs mutations counts are statistically significant, compared to other studies. Numbers in bars depict counts of altered samples. (**c**) Percentage of PCTAIRE alterations (including focal Copy Number Variations (CNV) and silent mutations) across cancer types. Most frequent genetic alteration of PCTAIREs is amplification, especially in UCEC, BRCA, OV, UCS and MESO. The most frequent type of mutation in PCTAIRE genes is missense mutation. (**d**) Graph reporting the mRNA expression levels of PCTAIREs across different cancer types (see Supplementary Table 2 for TCGA datasets acronyms meaning)

In order to explore the potential implications of PCTAIRE CDKs in tumor progression, we initiated a comparative analysis utilizing the Pan-TCGA dataset [96] (Supplementary Table 2 reports the studies utilized), as depicted in Fig. 4a. Globally, mutations affecting PCTAIREs have been observed in a variety of tissues and organs, spanning in 19 out of 33 TCGA cohorts, albeit with varying frequencies. Mutations on PCTAIRE genes mostly do not overlap, since tumors mutated in one of the three CDKs are generally not mutated for the other two (Fig. 4a). The prevalence of mutations in individual PCTAIRE genes is generally low, typically reaching around 1% for each of the three genes (Fig. 4a). Notably, among the 216 patients that carry PCTAIRE mutations, 148 (68.5%) are female and only 88 are (31.5%) male. In line with these data, the majority of PCTAIRE mutations are associated with cancers of the female reproductive system, such as ovarian (OV), endometrial (UCEC), and cervical (CESC) cancer. Conversely, no mutations in any of the PCTAIREs were identified in cancers of the male reproductive system, specifically prostate cancer (PRAD) (*n* = 498) and testicular germ cell tumor (TGCT) (*n* = 134). For non-reproductive organ and tissue cancers, there were 47 mutations in female and 88 in male patients.

**Fig. 5:**
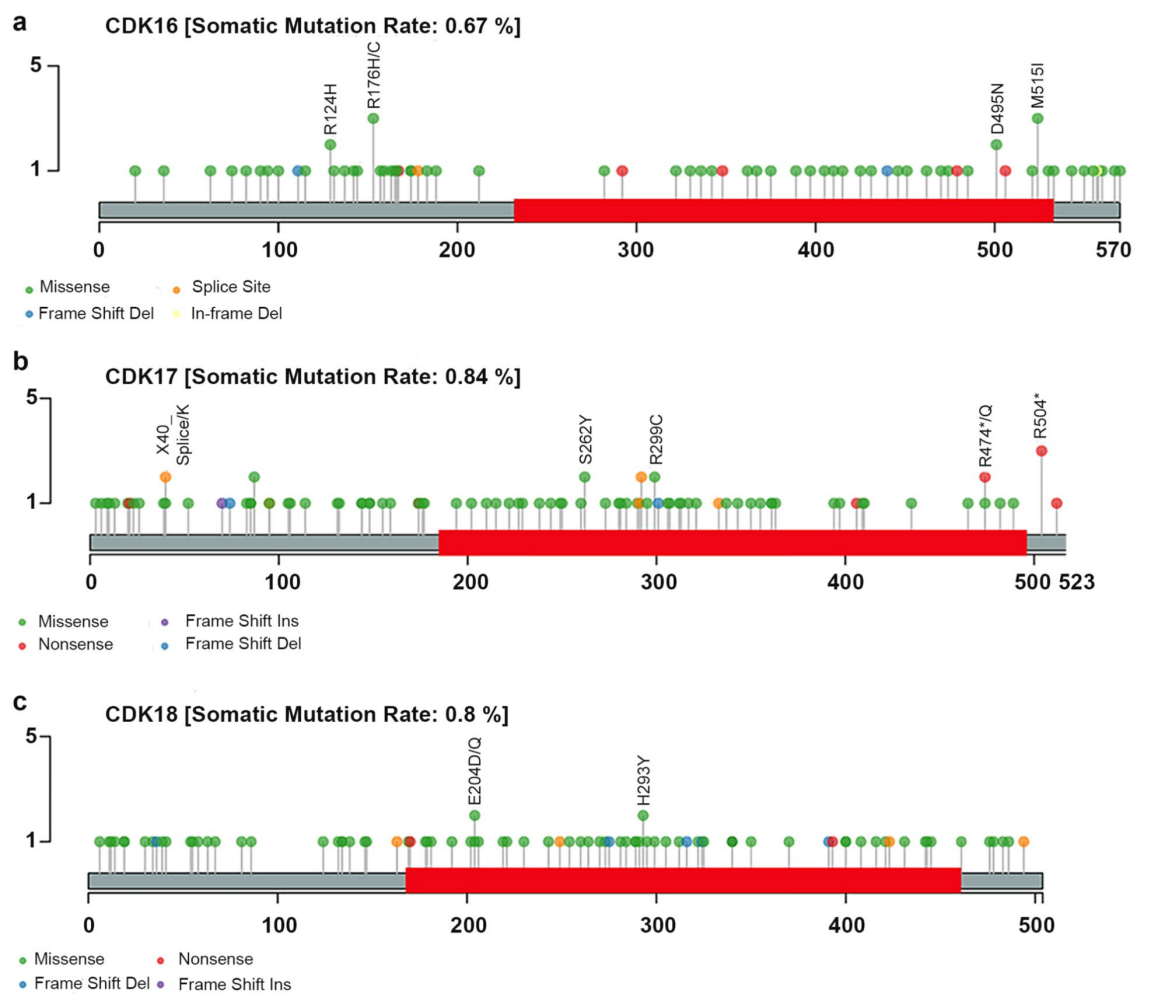
Pan-TCGA lollipop plots of PCTAIREs. TCGA mutation data was retrieved from GDC database [154]. Lollipop plots for PCTAIREs (**a**) CDK16, (**b**) CDK17 and (**c**) CDK18 are generated with Maftools package [155]. As shown, none of PCTAIREs present hotspot residue/location across cancer types. For CDK16, mutations in the residues 176 and 515 were observed two times. For CDK17, mutations in the arginine residue Arg504 (R504 in the plot) were observed in three cases. Lastly, CDK18 residue 204 was mutated in three cases

By conducting an analysis to identify recurrent mutations across the Pan-TCGA datasets [96], we established that there are no hotspots for mutation in any of the PCTAIRE genes, (see lolliplots graphs in Fig. 5). In the case of CDK16, the most frequently mutated positions were found at amino acid residues Arg176 and Met515 (Fig. 5a). CDK17 displayed mutations at Arg474 and Arg504 in three patients (Fig. 5b). Finally, CDK18 mutations were annotated three times at Glu204 and two times at His293 (Fig. 5c). Currently, no experimental studies reported possible functional consequences of these mutations, thus further evaluations on their possible roles and pathogenicity are needed. Mutations in all three PCTAIRE genes were significantly enriched in the UCEC datasets compared to other studies (*p* ≤ 0.01) (Fig. 4b).

Also, it was intriguing to observe the most frequent type of mutations that were occurring. Frameshift mutations are exceptionally rare in the CDK16, CDK17, and CDK18 genes across all the studies considered. The same trend is generally true for nonsense mutations, with the exception of CDK16 in Adrenocortical Carcinoma (ACC) (Fig. 4c). On the contrary, missense mutations were particularly prevalent compared to other alterations.

Uterine Endometrial carcinomas (UCEC) are the tumors most frequently mutated in PCTAIREs genes where ∼4% of analyzed samples carried missense mutations for CDK17 ∼3% for CDK18 and ∼2% for CDK16 of cases (Fig. 4c).

From transcriptomic studies on different tumor types, it is evident that PCTAIREs are highly expressed across the board (Fig. 4d). Notably, nearly in all studies, CDK16 exhibits higher expression levels compared to CDK17 and CDK18. The most prominent CDK16 expression is observed in renal chromophobe, ovarian and endometrial cancer (KICH, OV and UCEC), while the lowest levels were detected in leukemia (LAML). CDK17, on the other hand, shows its highest expression in Low-Grade Glioma (LGG) and CDK18 reaches its peak in renal clear cell carcinoma (KIRC). A more comprehensive transcriptomic comparison of PCTAIREs in normal and tumor conditions, using the Pan-TCGA datasets, is reported in Supplementary Fig. 9. In general, CDK16 is expressed at higher levels in tumors in about 60% of analyzed datasets, while CDK17 and CDK18 are overexpressed in 45% of them. Supplementary Fig. 9 reports the comparison between normal and cancer tissues for each tumor type. However, recognizing the biological implications of these differential expression levels of PCTAIREs across various tumors remains an uncharted field, in both basic and translational research.

### CDK16

Many studies have suggested a role for CDK16 in driving cancer cell proliferation through diverse mechanisms. One such mechanism involves the phosphorylation of p53 at the S315 residue by CDK16. This phosphorylation event prevents p53 nuclear localization and, conversely, leads to its degradation. Consequently, by inhibiting p53 activities, CDK16 promotes cell proliferation, survival, and radio-resistance [97]. Additionally, CDK16 has been shown to phosphorylate the cell cycle inhibitor p27Kip1 at Ser10, thereby destabilizing it and thereby promoting cell cycle progression, in transformed cells [98]. In accordance, it was shown that PLCβ1-mediated inactivation of CDK16, results in the rescue of p27Kip1 from CDK16 inhibition and, hence, in the block of cell cycle progression [99]. A study by Hernandez et al. further revealed that CDK16, in complex with CCNY, phosphorylates Protein Regulator of Cytokinesis (PRC1) at Thr481, inhibiting PRC1 nuclear localization and promoting cell proliferation [36]. Notably, this phosphorylation is particularly important during the S-G2/M phases of the cell cycle and is important for proper spindle formation in triple negative breast cancer (TNBC) cells [100]. As a consequence, pharmacological and genetic inhibition of CDK16 resulted in decreased TNBC cells growth in both in vitro and in vivo settings. Accordingly, CDK16 was found to be highly expressed in TNBC and its high expression predicts low patients’ survival [100].

Beyond its role in promoting cell proliferation, CDK16 is involved in regulating both autophagy and apoptosis. This dual role might be of particular significance in cancer biology, as autophagy can have both tumor-promoting and tumor-suppressive functions [101]. It has been shown that CDK16, in conjunction with CCNY, is involved in promoting autophagy in an AMPK-dependent manner [69]. Additionally, CDK16 regulates the extrinsic apoptosis pathway in prostate and breast cancer cell lines, contributing to cancer cell resistance to TNF-family cytokines, through the stabilization of RIPK1 [102]. Overall, these data support the possibility that CDK16 contributes to determine the balance between autophagy and apoptosis thereby driving cancer progression, as also proposed by others [78].

Accordingly, the reduction of CDK16 mediated by miR-494 may play a role in reducing the development of nasopharyngeal carcinoma [103]. Furthermore, in renal cell carcinoma, Fisetin treatment, a dietary tetrahydroxyflavone, was found to decrease the expression of both CDK16 and CCNY. This reduction was attributed to Fisetin’s ability to block the activity of Ten-Eleven Translocation protein 1 (TET1), thereby reducing proliferation and increasing apoptosis. It remains to be clarified whether CDK16 and CCNY act in concert or through distinct mechanisms [104].

CDK16 also appears to influence cell motility by modulating the JAK2/STAT3 pathway. Mechanistically, CDK16 mRNA acts as a sponge to bind and inactivate miR-324-5p, inhibiting its tumor-suppressive activity in hepatocellular carcinoma and melanoma cells [105, 106].

In conclusion, the available data support CDK16 as a potential biomarker for cancer diagnosis and prognosis. Furthermore, CDK16 may also represent a potential therapeutic target for specific tumor types that currently lack effective management strategies, as recently proposed [107]. Of course, more studies are needed to substantiate its oncogenic and potential therapeutic role(s).

### CDK17

Among all PCTAIRE family members, CDK17 is the least explored in the context of cancer, although some intriguing findings have already emerged. Bioinformatic analyses have highlighted CDK17 as one of the hub genes in glioblastoma, where its high expression is associated with better patient prognosis. This suggests a potential tumor-suppressive role for CDK17 in brain malignancies [108]. Furthermore, genetic data from TCGA datasets show that CDK17 exhibits focal copy number deletions, missense, and frameshift mutations in low-grade glioma (LGG) and glioblastoma (GBM), further supporting its potential as a tumor suppressor in brain cancers (Fig. 4).

However, CDK17 role(s) may differ in other cancer types. In pre-menopausal breast cancer patients, CDK17 appears to be overexpressed with a gradual increase from stage II to stage IV. Yet, the need for additional studies with larger cohorts and diverse technical approaches is essential to confirm this observation [109]. A recent multi-omic analysis, spanning over several cancer types, suggests that CDK17 is generally up-regulated in malignant tumors, associated with activation of epithelial-mesenchymal transition (EMT) and estrogen receptor pathways and with inhibition of apoptosis, cell cycle, and DNA damage response [110]. These findings seem to indicate that, with the exception of brain cancers, CDK17 may generally act as an oncoprotein in cancer. Nevertheless, experimental studies are required to validate this hypothesis.

In a study exploring the role of CDKs in the response to platinum chemotherapy in ovarian cancer CDK17 emerged as one of the CDKs necessary for survival. The study then primarily focused on CDK6, but the suggestion that CDK17 may play a role in platinum response certainly calls for further investigation [111].

To gain more insights into CDK17 potential functions in cancer, its expression levels in different cancer types were examined. CDK17 exhibits high expression in several tumor samples compared to their normal counterparts, including cholangiocarcinoma (CHOL), diffuse large B-cell lymphoma (DLBC), pancreatic adenocarcinoma (PAAD), and thymoma (THYM) [112].

Using these datasets, we retrieved genes whose expression were positively correlated with CDK17 expression (Spearman correlation coefficient > 6) [113]. As depicted in the Venn diagram, 5 genes commonly correlated with CDK17 expression in all 4 datasets (NEDD1, PPP1R2A, MED13L, ELK3, QKI) (Supplementary Fig. 10). MED13L activates transcription for most RNA polymerase II-dependent genes [114]. ELK3, when phosphorylated by ERK1/2, acts as a transcriptional activator and is involved in epithelial-mesenchymal transition, cell adhesion, migration, and angiogenesis [115]. Interestingly, a positive correlation between CDK17 and ELK3 expression has been observed also in neurons after axotomy [116]. NEDD1 ensures proper microtubule nucleation during the G2/M transition and is phosphorylated by PLK1 [117]. PPP1R12A is involved in de-phosphorylation of PLK1, which can lead to mitotic arrest [118]. Finally, QKI belongs to the STAR family of RNA binding proteins, regulating mRNA splicing, turnover, and stability and the abundance of circular RNAs, with implications in epithelial-mesenchymal transition [119, 120]. The connection between CDK17 and QKI is further supported by the finding that circular RNA produced at the CDK17 locus (circCDK17) is overexpressed in cervical cancer, and it functionally regulates disease progression [121, 122]. It is important to note that CDK17, ELK3 and NEDD1 are located within the same chromosomal region (Chr12 q23.1). Therefore, it is important to understand (1) whether abovementioned co-expression patterns are merely due to genomic amplification/deletion events, (2) if MED13L impacts on their expression in a similar manner, due to its transcriptional role (3) last but not least, whether CDK17 engages with these proteins, to exert any particular biological function.

### CDK18

Relatively more studies have attempted to address the role of CDK18 in cancer progression, compared to CDK17. Initial indications of CDK18 potential involvement in tumor progression emerged from studies showing that silencing CDK18 inhibited the growth of cutaneous T-cell lymphoma cells, suggesting that CDK18 may play a role in promoting their growth [123]. However, the effects of CDK18 in different cancer types can vary. For instance, in glioma cell lines, CDK18 expression was induced by treatment with the p53-derived chimeric analog, known as Chimeric Tumor Suppressor (CT-1). Interestingly, this induction of CDK18 led to growth arrest and cell death in the glioma cells [124]. This implies that CDK18 role may be context-dependent, and its effects on cancer progression can differ based on the specific type of cancer and microenvironmental conditions.

In another study, CDK18 has been shown to negatively modulate Focal Adhesion Kinase (FAK) activation, through direct cytoplasmic phosphorylation, using Hela cells. This suggests that CDK18 may play a role in regulating cell adhesion and migration, processes often associated with tumor spreading [125].

CDK18 has also been implicated in maintaining genome stability and DNA damage response. Depletion of CDK18 in colorectal cancer cells resulted in increased DNA damage and stalled replication fork, when exposed to replicative stress [126]. The induction of replication stress is largely due to the activation of ATR signaling pathway [127]. Interestingly, CDK18 also plays a role in ATR-mediated Homology-Directed Repair (HDR), in glioblastoma. CDK18 knockdown or ATR inhibition, in glioblastoma stem-like cells (GSCs), suppressed HRD and conferred PARPi sensitivity [128]. This suggests that CDK18 expression could serve as a biomarker for determining the administration of PARP inhibitor therapy, especially when other biomarkers of homologous recombination deficiencies are not available.

Similar data have been observed in breast cancer, where high levels of CDK18 were associated with increased sensitivity to replication stress-inducing chemotherapeutic agents. High CDK18 protein expression was linked to the basal subtype of breast cancer and improved patient survival in estrogen receptor (ER)-negative breast cancers treated with chemotherapy [129].

CDK18 has been reported to be overexpressed in gastric cancer, where it appears to promote cancer cell proliferation and reduce T cell tumor infiltration [130]. Finally, transcriptome and proteome analyses of pituitary adenomas revealed upregulation of CDK18 in gonadotropes and null cell adenomas. However, functional data regarding its role in these tumors are currently lacking [131].

Overall, the available data suggest that CDK18 has multifaceted functions in human cancer and can act either as oncogene or as tumor suppressor, depending on the specific tumor type and context. Further research is needed to fully understand the complexities of CDK18 roles in different cancer types and determine its potential as a biomarker and/or therapeutic target.

## Future perspective and open questions

Here, we have completed a comprehensive review of the literature and of available databases to summarize all main features and possible roles of the understudied PCTAIRE CDKs in normal tissues and in cancer emerges quite clearly that PCTAIREs research is still in its infancy. However, as witnessed here and proposed by others [132], these CDKs hold great promises to expand our knowledge about organ and cellular physiology and the pathogenesis of different diseases.

To summarize, we report the key points that stand out and certainly deserve further consideration:

### Structure and activation

The unique structural features and activation mechanisms of CDK16, CDK17, and CDK18, set them apart from other CDKs. Their N-terminal extensions, distinct phosphorylation requirements, and activation by cyclins make them intriguing subjects for future investigation. Understanding the precise mechanisms of their activation will be essential. In this context it is worth considering that PCTAIREs evolved from the ancestral CDK5, which is quite unique in its activation regulation. Indeed, CDK5 could be activated by the association with p35^nck5a^ or by its truncated form, p25^nck5a^ [133, 134] and with p39^NCK5AI^, which are not typical cyclin proteins. Both p35 and p39 are myristoylated and enriched at the plasma membrane in neurons due to their signal, leading to consequent membrane recruitment of CDK5 [135]. Interestingly, p25 lacks the myristoylation signal, causing CDK5 hyperactivation and aberrant cellular localization, suggesting that the kinase activity of CDK5-p35 is regulated through its association with the membrane [136, 137]. Based on these evidences, it would be probably necessary to look for a new family of CDKs activating proteins that differ from classical cyclins for their localization, regulation and binding domains. Structural data, mainly from Musacchio lab [138, 139], indicate that p35 and p25 lack the tandem copies of the cyclin-box fold (CBF) to bind CDKs and have instead a single CBF-like motif (CBFL) that is necessary for their interaction with CDK5. We believe that PCTAIREs could either be activated by proteins containing a CBFL motif or that they might have a basal kinase activity even in the absence of a regulatory subunit. Studying the interactome of PCTAIREs in different context and cell types, for instance by Mass Spectrometry unbiased approaches, could help in identifying possible regulatory subunits and/or ways of activation.

Another striking difference between CDK5 and mitotic CDKs (e.g. CDK1 or CDK2) is the opposite role of their phosphorylations. The phosphorylation at Tyr15 by Abl is responsible for the stimulation of CDK5 activity, while phosphorylation of Tyr15 and Thr14 by Wee1 family of kinases is inhibitory for CDK1 and CDK2. Ser159 phosphorylation on CDK5 is responsible for the inhibition of its kinase activity, while CDK2 requires the phosphorylation on a similar a threonine residue (Thr160 in CDK2) by the CDK-activating kinase (CAK) for its maximal activation. Accordingly, CDK5 is not phosphorylated by CAK [138, 140–143]. Overall, these data support the possibility that also PCTAIREs’ activity is differently regulated by phosphorylation events and suggest that identifying possible Tyr and/or Ser/Thr kinases able to interact with and phosphorylate the three PCTAIREs would support a better understanding of their regulation.

Finally, we want to highlight here that p35 and p25 participate in substrate recognition and specificity of CDK5, supporting the possibility that, also for PCTAIREs, activator subunits could contribute to define which are the preferential substrates. Of course, these substrates could be extremely diverse considering normal and pathological conditions and different subcellular localizations.

### Subcellular localization

The distinctive subcellular localization of PCTAIRE CDKs in the cytoplasm and at the plasma membrane, often in association with cytoskeletal elements, suggests roles in vesicular transport, adhesion, and cell motility. The need for discovery of the exact domains responsible for interactions with the cytoskeleton warrants further study. Moreover, the evidences that connect PCTAIREs with the control of nuclear localization and typical nuclear functions, like cell proliferation and DNA repair, merit to be better confirmed. We can speculate that, as evidenced for CDK5, the association of PCTAIREs to plasma membrane could be driven by their regulatory subunit. Indeed, CDK5-p35 or CDK5-p39 complexes are localized at plasma membrane, since both p35 and p39 are myristoylated [135]. The CDK5-p25 complex is localized in the cytoplasm, since p25, a cleaved product of p35, does not contain the myristoylation signal. Membrane localization of CDK5 is also associated with its decreased kinase activity. These evidences suggest that, also for PCTAIREs, subcellular localization could impact on their activation, substrate specificity and, eventually, their functions. Generation of better and more specific research tools (e.g. antibodies, conditional mouse models, CRISPR/Cas9 mediated cellular knock out, specific inhibitors, etc.) would be very important to expedite the proper dissection of PCTAIREs subcellular localization and function, in normal and transformed cells.

### Neuronal and psychological disorders

Initial research has primarily focused on the role of PCTAIRE CDKs in terminally differentiated neural cells and their implications in various neurological and psychological disorders. Expanding these investigations to include animal models and embryonic development will provide a more comprehensive understanding.

One really surprising observation was the completely different phenotypes reported for the knock-out of CDK16, CDK17, CDK18 in mice, ranging from mild defects in specific tissue/organs to a possible gender specific embryonic lethality for CDK17. Since these data are still largely unpublished they should be taken with caution. Yet, if confirmed in more thorough studies, understanding what is the specific role(s) of CDK17 during embryogenesis and why its absence is more relevant in females than in males would be of extreme relevance. The fact that the analyses of human tumors indicate that PCTAIRE mutations are more common in female than in males and usually found in cancers of the female reproductive system, such as ovarian (OV), endometrial (UCEC), and cervical (CESC) cancer, support a gender specific role for these CDKs and, especially, for CDK17.

### Interplay in cancer

Recent studies have started to reveal more details on the alterations and possible roles played by PCTAIRE CDKs in cancer. They appear to have both overlapping, particularly in cell cycle regulation, apoptosis, and DNA-damage repair pathways, and distinct functions, overall highlighting the need for further clarification of their individual contributions and potential crosstalk. Investigating their roles in autophagy and vesicle transport in different cancer settings, particularly those in the genitourinary system, holds promise. One completely unexplored area is the role of PCTAIREs during tumor progression and in response to therapies. Do their expression, mutational status and/or copy number, change in metastatic compared to primary tumors or after treatment with specific drugs? If yes, which could be their role in these settings? Could subclonal alterations in PCTAIREs drive tumor clonal evolution or they only represent passenger alterations associated with tumor progression? These questions are of primary relevance, especially in the context of those tumors, like the ones of the female reproductive system, probably the most affected by PCTAIRE alterations. In case some causal correlation will be found, it could be speculated that their pharmacological inhibition could be used to prevent tumor recurrences or improve the efficacy of standard treatments. Only well designed translational studies will be able to respond to these important questions.

### Clinical implications

The possibility of generating specific inhibitors for PCTAIRE CDKs opens the way for clinical translation (this manuscript and a briefing by Axtman et al.,) [132]. These inhibitors could have therapeutic relevance in neurodegenerative diseases, like Alzheimer’s, and as anticancer agents. This research area represents a significant opportunity to address unmet medical needs in both fields. At this regard, it is worth mentioning that we do not know how exactly PCTAIREs could be activated and, consequently, we can only speculate on how they could be inhibited, based on analogous studies that addressed this issue mostly in mitotic CDKs. As mentioned above, it is more than possible that PCTAIRE kinase activity is regulated in a completely different manner compared to mitotic CDKs, raising the doubt that different approaches should be pursued to specifically target these forgotten CDKs. We firmly believe that these approaches, aimed at identifying, developing and optimizing high-quality drug candidates for novel targets, like PCTAIREs, in the oncology field, should be based on the integration of biology, chemistry, and data science, as recently proposed [64].

In summary, by the completion of this review we have underscored a great potential of PCTAIRE CDKs, in advancing our understanding of cell biology, physiology, and the pathogenesis of various diseases. By delving deeper into their structural peculiarities, activation mechanisms, and roles in normal and disease states, we may be able to identify unique contexts for future therapeutic development and clinical applications.

### Electronic supplementary material

Below is the link to the electronic supplementary material.

Supplementary Material 1

## Data Availability

No datasets were generated or analysed during the current study.

## Abbreviations

Cables1: Cdk5 and Abl enzyme substrate 1
CATDS: Concentration And Target Dependent Selectivity
CCNY: Cyclin Y
CCNYL1: Cyclin Y-like 1
CDK: Cyclin-Dependent Kinase
CT-1: Chimeric Tumor Suppressor 1
DKK: Dark Kinase database
DYRK: Dual-specificity Tyrosine-Regulated Kinase
EMT: Epithelial-Mesenchymal Transition
ER: Estrogen Receptor
FAK: Focal Adhesion Kinase
GSK3β: Glycogen Synthase Kinase-3 beta
GWAS: Genome-Wide Association Study
HDR: Homology-Directed Repair
IMPC: International Mouse Phenotyping Consortium
MAPKs: Mitogen-Activated Protein Kinases
MBP: Myelin Basic Protein
MSA: Multiple Sequence Alignment
PRC1: Protein Regulator of Cytokinesis
TET1: Ten-Eleven Translocation protein 1
TNBC: Triple Negative Breast Cancer
TRAP: Tudor repeat associated with PCTK2 (Note List of abbreviations for TCGA datasets is retrievable in Supplementary Table 2)
