# Supplementary material for: Insights into the structural and functional activities of forgotten Kinases: PCTAIREs CDKs
Source: Mol Cancer. 2024 Jun 29;23:135. doi: 10.1186/s12943-024-02043-6 (PMC11218289; doi:10.1186/s12943-024-02043-6)
Supplement: Supplementary file 1 — Supplementary Material 1 [file 12943_2024_2043_MOESM1_ESM.pdf]

Insights in structural and functional activities of forgotten Kinases: PCTAIREs CDKs

Javad Karimbayli, Ilenia Pellarin, Barbara Belletti and Gustavo Baldassarre

Supplementary material

Contents

**Supplementary Figure 1**	Related to Figure 1
**Supplementary Figure 2**	Related to Figure 1
**Supplementary Text**	Related to Figure 2 and paragraph 3
**Supplementary Figure 3**	Related to Figure 2
**Supplementary Figure 4**	Related to Figure 2
**Supplementary Figure 5**	Related to Figure 2
**Supplementary Figure 6**	Related to Figure 2
**Supplementary Figure 7**	Related to Figure 2
**Supplementary Figure 8**	Related to paragraph 5
**Supplementary Figure 9**	Related to paragraph 7.1
**Supplementary Figure 10**	Related to paragraph 7.2
**Supplementary Table 1**	Related to paragraph 6
**Supplementary Table 2**	Related to Figure 4 and Supplementary Figure 9

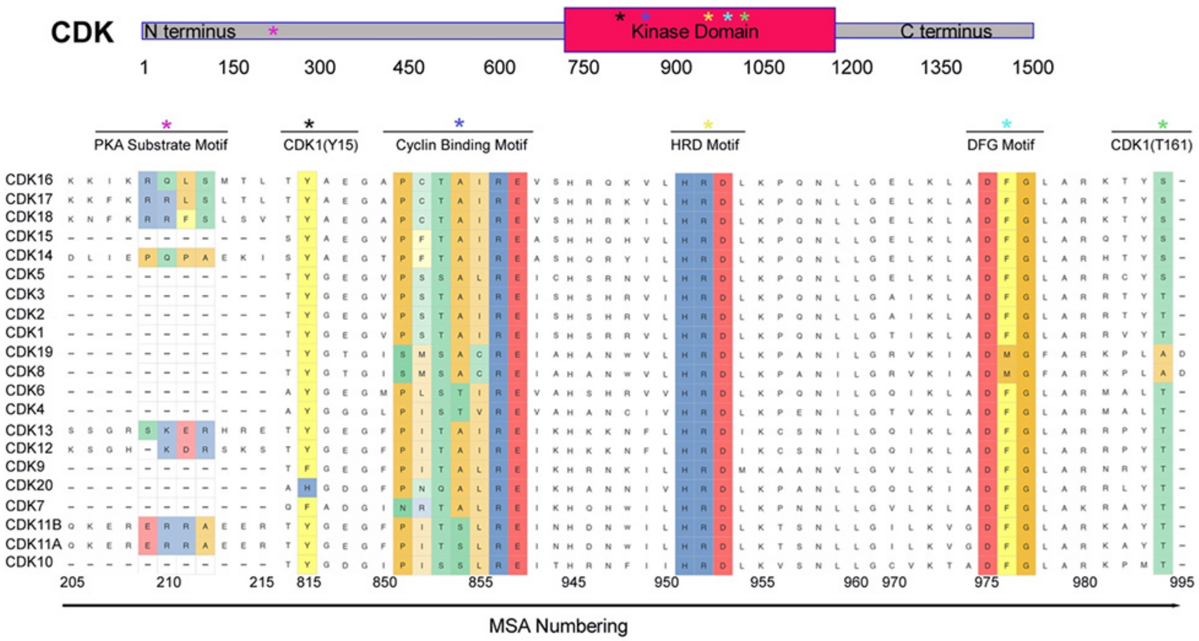

Supplementary Figure 1. Multiple Sequence Alignment (MSA) of human CDKs. Graphical representation of conserved domains among human CDKs. MSA numbering refers to the alignment position. Region of interests are evidenced: purple asterisk marks the PKA substrate motif, located in the PCTAIRE N-terminal domain; black asterisk marks the conserved tyrosine residue, in the kinase domain (homologous to Y15 of CDK1, a known phosphorylation site with inhibitory role); blue asterisk marks the cyclin binding motif, in the kinase domain; yellow asterisk marks the HRD motif, in the kinase domain; sky-blue asterisk marks the DFG motif located in the kinase domain; green asterisk marks the conserved threonine/serine residue, in the kinase domain (homologous to 161Y of CDK1, a known phosphorylation site with activating role). Color code is used according to the chemistry of amino acids. Sequences were aligned and visualized with GGMSA software (1).

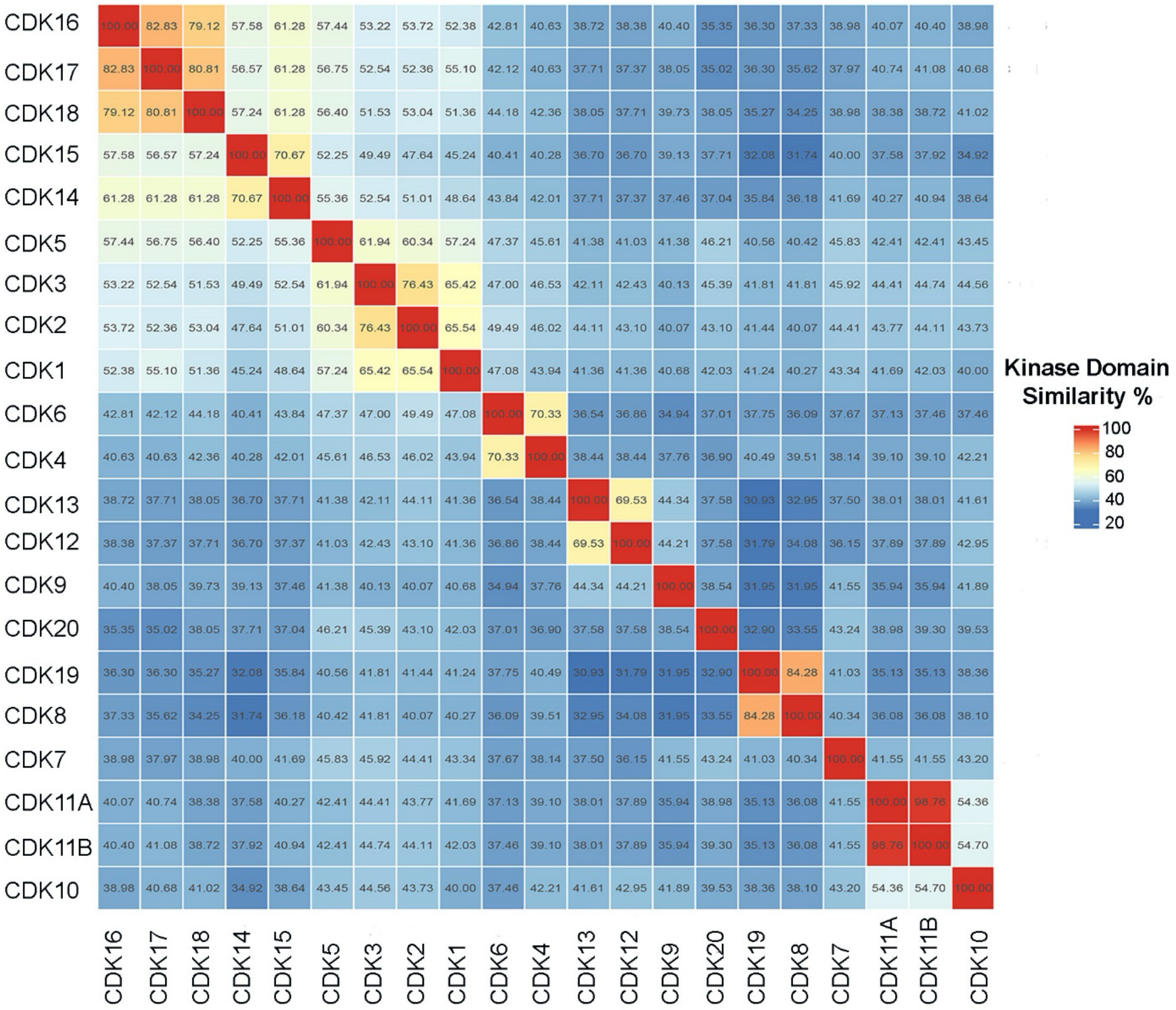

Supplementary Figure 2. Similarity among the kinase domains of the 21 human CDKs. Heatmap table reporting the graphical representation of sequence similarity among the kinase domains of the 21 human CDKs. The sequence of kinase domain of each CDK has been compared to all the other sequences and the similarity scores was calculated with SeqInr (2) and then plotted with Complex Heatmap package (3).

Supplementary Text, Related to figure 2 and paragraph 3

PCTAIRE 3D-Structure Analysis

Detailed structural and mechanistical studies on atypical CDKs are scarce. However, computational tools could aid in generating and then test specific hypothesis (4,5). Here, we used the computational tool developed by McSkimming. D et.al, to predict active/inactive state of kinases, based on their 3D-configurations (6).

We compared the CDK16 3D-structure with the ones of CDK1, CDK2 and CDK5, which are the closest homologues to PCTAIREs among CDKs for which experimental structure was available. Corresponding PDB files were retrieved from Protein Data Bank database.

Obtained CDKs structures were annotated as either active or inactive, based on McSkimming D et al. (6). In total, we annotated 365 PDB files for CDK1, CDK2, CDK5 and CDK16 (either curated or predicted). CDK16 have two deposited structures in protein databank [PDB-ID: 5G6V_A and 3MTL_A]. We used the CDK16 [PDB-ID: 5G6V_A] that, like the other available CDK structures used, displays the cyclin binding domain in a helical conformation. Most of the annotated structures belong to CDK2. For CDK1, we had two PDBs: in active [PDB-ID:4Y72_A] and inactive [PDB-ID: 4YC6_A] conformation. All available CDK5 structures available were in active configuration, either annotated or predicted.

Annotated PDB files were used as input for multiple structure alignment with the local version of mTM-align software (7) with default settings at GARR (Italian National Computer Network for Research and Universities) high performance computer. Further, visual inspection and comparison of structures were performed with Chimera software (8), as illustrated in (Supplementary Figure 3a). Briefly, mTM software outputs two scoring functions: 1) TM-score (Template Scoring), a length-independent scoring function, which is a robust metric for overall structural similarity (score 1 means identical structures); 2) RMSD (Root Mean Square Deviation), a metric for local

similarities in protein structures (lower RMSD reflects better local similarities between proteins). Both TM and RMSD scores gave similar results among active and inactive structures and, based on these scores, CDK active and inactive structures clustered separately (Supplementary Figure 3b, c). To our surprise, the predicted inactive 3D-structure of CDK16 clustered with the active structures of CDK1, CDK2 and CDK5 (Supplementary Figure 3b, c). Visual inspection of conserved regions revealed a high degree of conservation at the core of CDK kinase domains (Supplementary Figure 3d; conserved regions, colored in blue, are mapped on CDK16 structure). A deeper look into the specific motifs known to govern CDK activation (*i.e.*, cyclin binding domain, DFG, HRD and CDK16 specific additional insertion in CDK/MAPK domain (9) (hereafter referred to as MAPK domain) revealed that only HRD is highly conserved among the analyzed CDKs. The residues flanking the cyclin binding, DFG and the MAPK motifs (but not themselves) are similarly well conserved (Supplementary Figure 3e).

Since hierarchical clustering revealed that inactive CDK16 structure is more similar to active than inactive CDK1, CDK2 and CDK5 structures, we looked for similarities/differences between CDK16 and the other CDKs in the motifs that undergo reconfiguration upon cyclin binding (*i.e.* cyclin binding domain, HRD, DFG). To this aim, we visually compared CDK16 to CDK1, CDK2 and CDK5 structures, as detailed below.

First, heatmap analyses confirmed that CDK16 had relatively higher TM and lower RMSD scores with active CDK1 and CDK2 structures, compared to inactive ones (Supplementary Figure 4a, b). Pairwise comparison of CDK16 with active and inactive CDK2 confirmed that it has relatively high global similarity with active CDK2 configuration (Supplementary Figure 4c). The cyclin binding motif of CDK16 (PCTAIRE) is slightly more similar to active CDK2 cyclin binding motif (PSTAIRE), compared to the inactive one (Supplementary Figure 4c, inset a). The DFG motif of

CDK16 does not overlap with the corresponding motif of CDK2 structure, while the topology of the HRD motif is quite similar comparing CDK16, with both active and inactive CDK2 structure. Similarly, the active CDK1 structure [PDB-ID: 4Y72] shares the lowest RMSD score with CDK16 (Supplementary Figure 4d) and, accordingly, CDK16 displays a better overlap with active than with inactive CDK1 3D-configuration (Supplementary Figure 4d). Moreover, the PCTAIRE motif shows better spatial overlap with the PSTAIRE motif of active than inactive CDK1 (Supplementary Figure 4d, inset 1). Differently from CDK2, the active CDK1 cyclin binding domain overlaps well with the corresponding domain of CDK16 (compare Supplementary Figure 4c inset1 with 4d inset1). Finally, while the DFG motif of CDK16 is topologically different from that of CDK1 (Supplementary Figure 4d, inset 2), the HRD motifs of CDK16 and active/inactive CDK1 structures are topologically highly similar to each other (Supplementary Figure 4d, inset 3). Overall, these comparative analyses revealed a high degree of 3D similarity of inactive CDK16 with active CDK1 and CDK2 structures.

Next, we compared CDK16 to CDK5, the archetype of non-canonical CDK, observing a high degree of overall similarity between the 3D-structures of the two CDKs. Of note, all CDK5 structures annotated in PDB are deposited in active configuration and the cyclin-binding motifs of CDK16 and CDK5 have different 3D-coordinates (Supplementary Figure 5, inset 1). Additionally, DFG motif of CDK16 is spatially out of the coordinates of the corresponding CDK5 motif (Supplementary Figure 5, inset 2). Last, topology of HRD motif of CDK5 is similar to the one of CDK16 (Supplementary Figure 5, inset 3). Overall, these data suggested that the cyclin binding domains of CDK16 is topologically more similar to the corresponding domain of active CDK1.

Next, we compared CDK5 to CDK1 and CDK2 structures. TM and RMSD scores of CDK5 structures are relatively higher for active CDK1 and CDK2 structures compared to inactive ones (Supplementary Figure 6). We used CDK5 structures [PDB-IDs: 4AU8 and 1UNH], which have

the highest TM (0.881) and the lowest RMSD (1.995), compared to CDK16. In general, CDK5 presents good similarity with active CDK1 and CDK2 structures (Supplementary Figure 6). CDK5 cyclin binding domain is spatially overlapping with active CDK2, but not with active CDK1 cyclin binding motif (Supplementary Figure 6, inset 1). DFG and HRD motifs of CDK5 are similar to both active CDK1 and CDK2 corresponding motifs (Supplementary Figure 6, inset 2 and 3, respectively). As mentioned, no curated or annotated CDK5 structures are categorized as inactive and, consequently, we were not able to distinguish configurational differences between active and inactive CDK5 structures with respect to CDK1 or CDK2.

Lastly, we evaluated whether the observed features, particularly the similarity in cyclin binding motif of CDK16 with active CDK1, are unique to CDK16 or shared among other PCTAIREs subfamily members.

Since CDK17 and CDK18 structures are yet to be experimentally determined, using the SWISS-Modell tool (10) we modelled CDK17 and CDK18 structures, using CDK16 structure as template [PDB-ID: 5G6V] and then visually compared them to CDK16. This approach revealed a high degree of shared geometry among the PCTAIRE family members (Supplementary Figure 7). We observed small differences at PCTAIRE and DFG motifs, between CDK18 *in silico* structures and the others (Supplementary Figure 7, inset 1-3). In particular, the side chain of phenylalanine of CDK18 DFG motif is notably different, compared to CDK16 and 17. This observation is in line with the structure-guided alignment results, which showed that similarity is comparatively higher between CDK16 and CDK17 (72.5 %) than between CDK16 and CDK18 (65.7 %) or CDK17 and CDK18 (69.7%) (see main text, Figure 1c and Supplementary Figure 2).

Overall, these analyses revealed that the structure of CDK16 in an inactive configuration is surprisingly more similar to the active than the inactive 3D-structures of CDK1, CDK2 and CDK5. Of note, the cyclin binding domain of CDK16 overlaps very well with the corresponding motif of

active CDK1, possibly suggesting that understudied PCTAIRE CDKs may differ from canonical CDKs in their activation mechanisms.

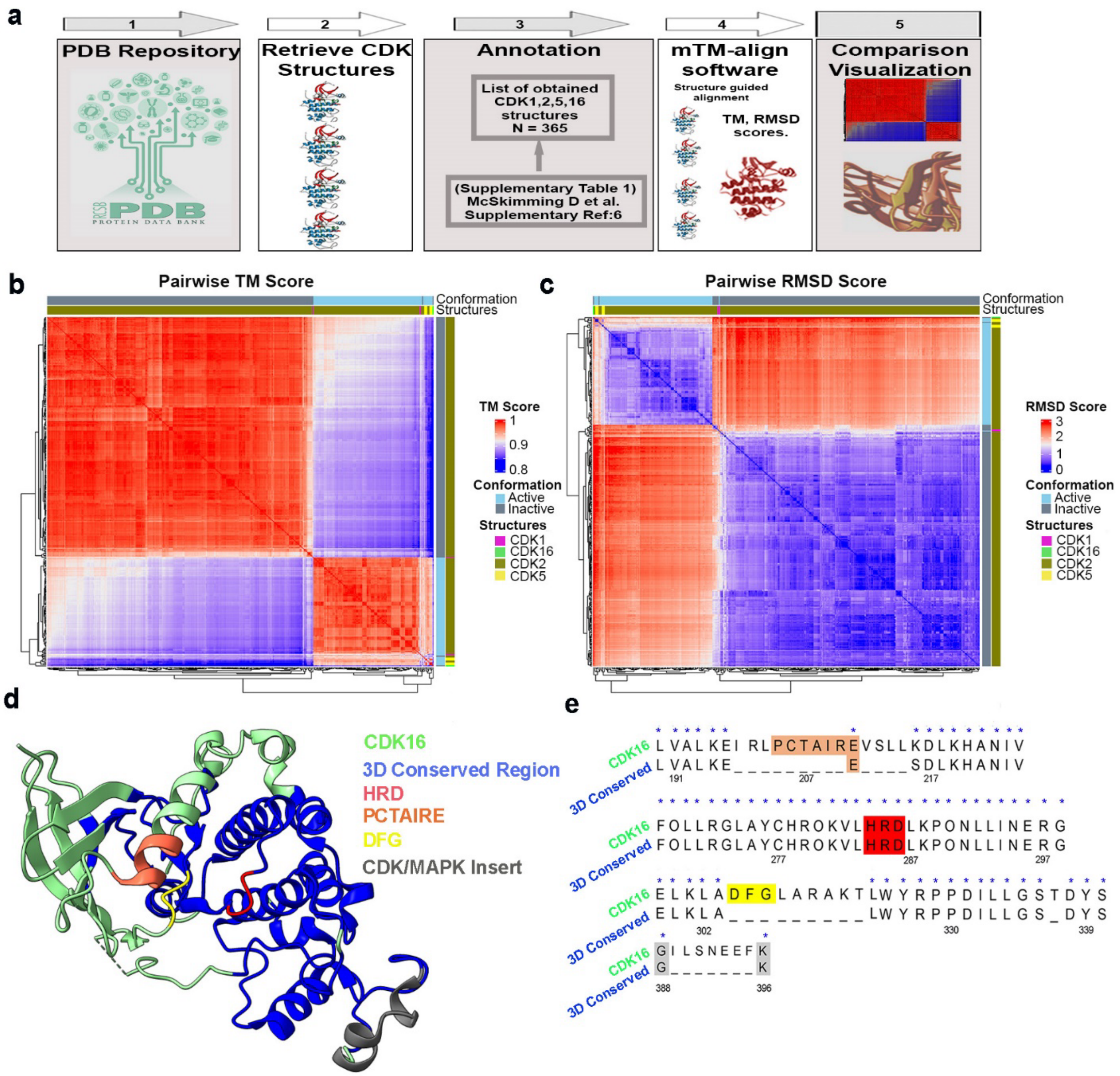

Supplementary Figure 3. Pairwise comparison of annotated CDK structures. Multiple structure alignment of CDK16 [PDB-ID: 35G6V_A] with annotated structures of CDK1, CDK2 and CDK5. **a)** Workflow diagram illustrating step by step analysis**. b)** and **c)** Heatmaps displaying Pairwise TM and RMSD scores. Obtained structures clustered according to the kinase conformation status (active, *versus* inactive). **d)** 3D view of CDK16 structure. Blue regions represent conserved regions among CDK16 and CDK1, CDK2 and CDK5. HRD, DFG, PCTAIRE and CDK/MAPK insert regions are highlighted in red, yellow, orange and gray, respectively. Only the HRD motif is

comprised among the conserved regions. Visualization was obtained with Chimera software (8). **e)** 2D view of CDK16 sequence and conserved regions, depicting PCTAIRE, HRD and DFG-spanning regions of CDK16 protein, visualized with Bioconductor package ggmsa.[1] PCTAIRE and DFG motifs are not conserved among CDK16 and CDK1, CDK2 or CDK5 structures, but their spanning residues are conserved. Different from PCTAIRE and DFG motifs, the 3D-coordinates of HRD motif, as well as its spanning region, are conserved. Asterisks mark conserved residues.

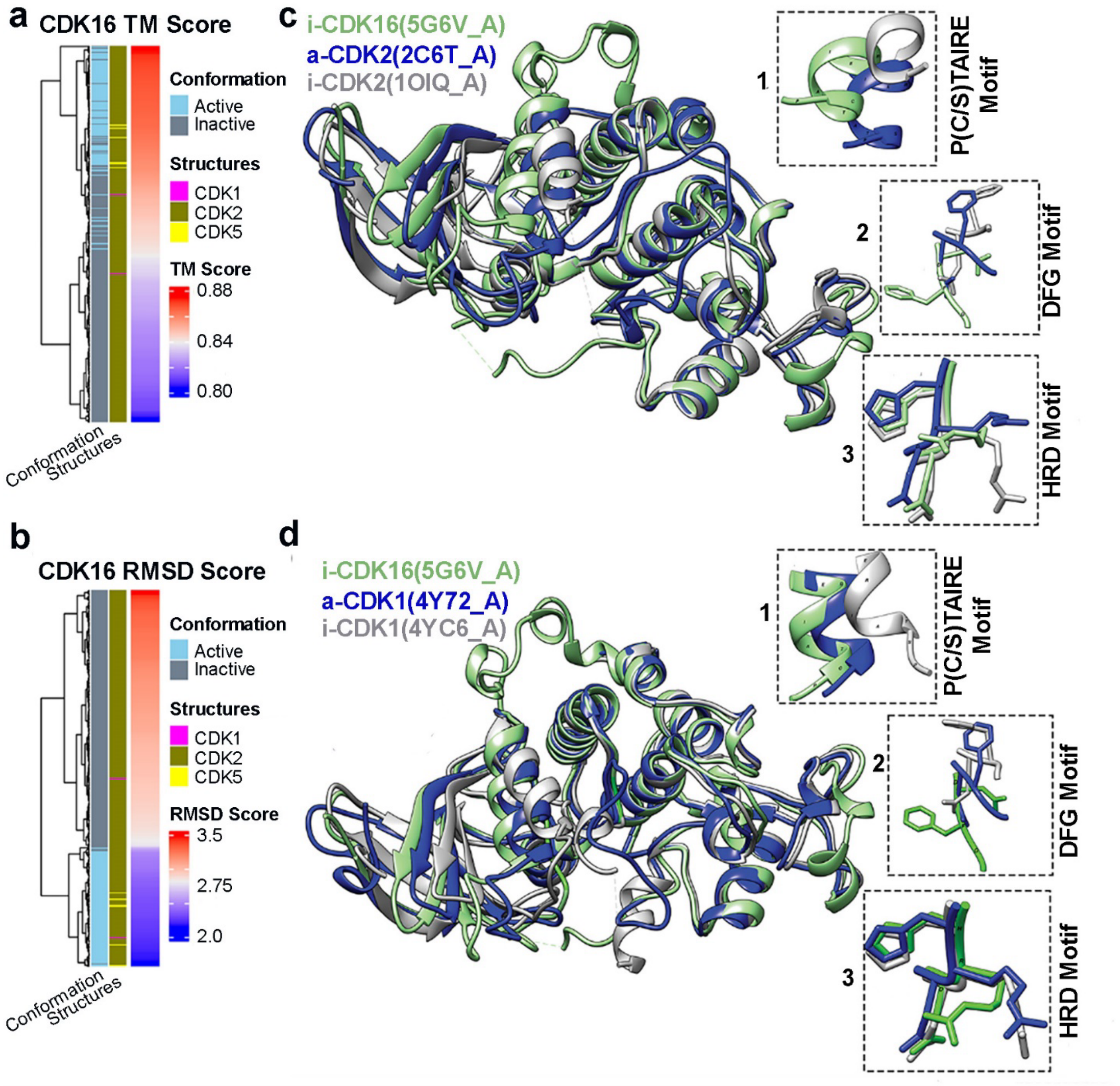

Supplementary Figure 4. Inactive CDK16 shares high similarity with active CDK1-2 structures, a) and **b)** Heatmap representation of TM and RMSD scores of CDK16 *versus* CDK1-2-5. **c)** CDK16 structure was superimposed with active CDK2 [2CTA_A] and inactive CDK2 [PDB-ID: 1OIQ_A] in Chimera.(8) **Insets 1, 2, 3** are zoom-in view

of PC(S)TAIRE, DFG and HRD motifs. **d)** CDK16 structure was superimposed with active CDK1 [PDB-ID: 4Y72_A] and inactive CDK1 [PDB-ID: 4YC6_A]. **Insets 1, 2, 3** are zoom-in views of PC(S)TAIRE, DFG and HRD motifs. As shown, cycling binding motif of CDK16 has very close spatial coordinates to the respective motif of the active CDK1. DFG motif of CDK16 is out of the CDK1 and 2 DFG-motif planes.

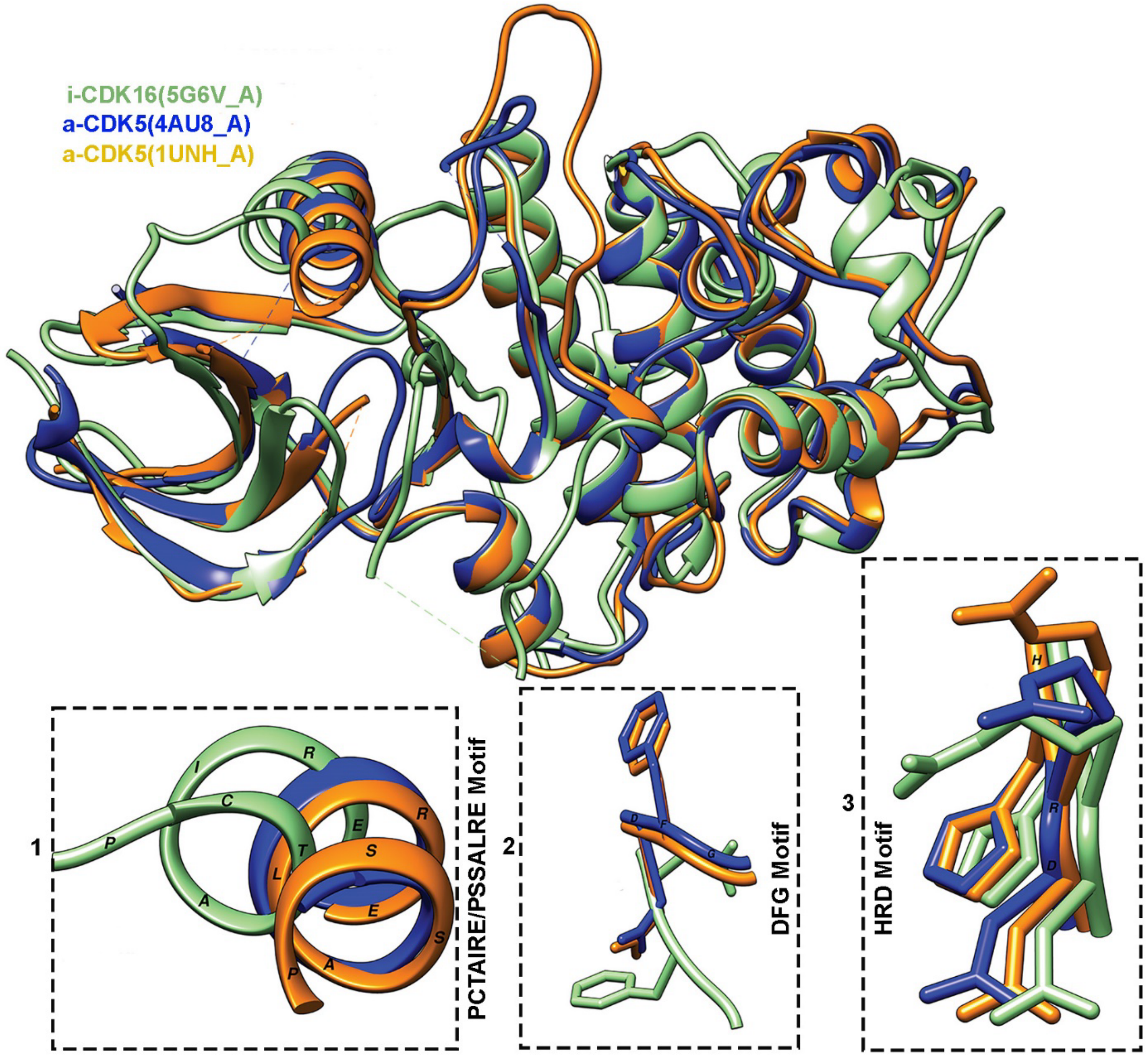

Supplementary Figure 5. Superimposition of CDK16 structure with CDK5. CDK16 structure was superimposed with active CDK5 [PDB-ID:4AU8_A] and CDK5 [PDB-ID:1UNH_A] structures to comparatively visualize topological similarities/differences. Superimposition and visualization were performed as described earlier (see footnote Supplementary Figure4). **Insets 1, 2, 3** are zoom-in views of PC(T)AIRE, DFG and HRD motifs, respectively.

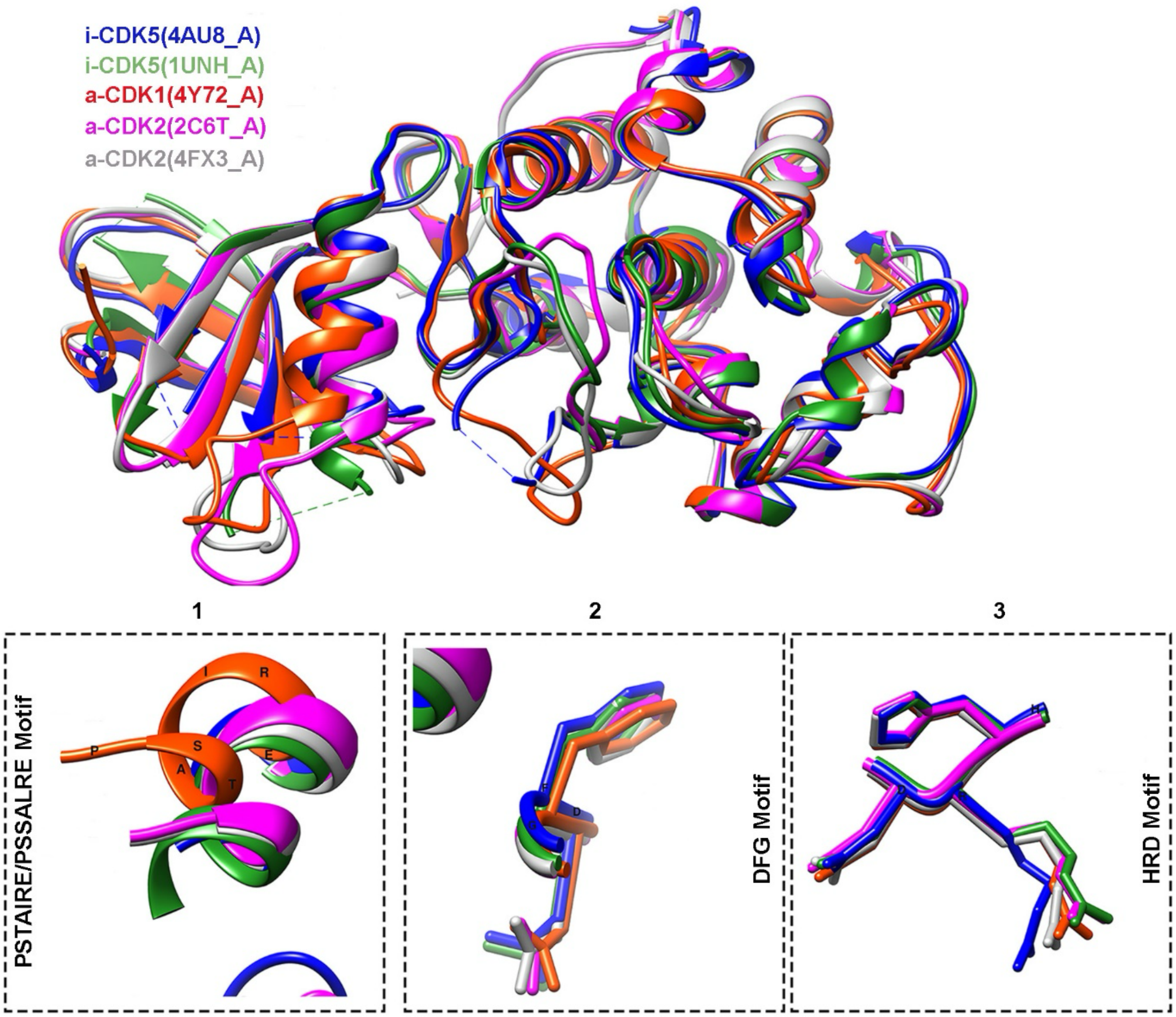

Supplementary Figure 6. Pairwise comparison of CDK5 structures with CDK1 and 2. Superimposition of the indicated CDK5 structures with CDK1 and CDK2 was performed as described earlier (see footnote Supplementary Figure4). Like CDK16, CDK5 [PDB-ID: 4AU8-A] had lowest RMSD score respect to CDK1 [PDB-ID: 4Y72_A]. CDK2 structure [PDB-ID: 4FX3] presents the highest TM-score compared to CDK5 [PDB-ID:4AU8_A]. In order to better compare CDK16 and CDK5 in regard to active CDK2, we also included CDK2 in our analysis [PDB-ID: 2CT6-A], since it presented the highest TM and lowest RMSD scores compared to CDK16 (Supplementary Figure 2a and b). CDK5 shares overall high similarity with both active CDK1 and CDK2. More in detail, CDK5 cyclin binding motif overlaps better with the one of CDK2 than with the one of CDK1 (**inset 1**). Differently from CDK16, CDK5 DFG motif is on the same plane of both CDK1 and CDK2 DFG motifs (**inset 2**). The HRD motif of the 3 CDKs is also well topologically conserved (**inset 3**).

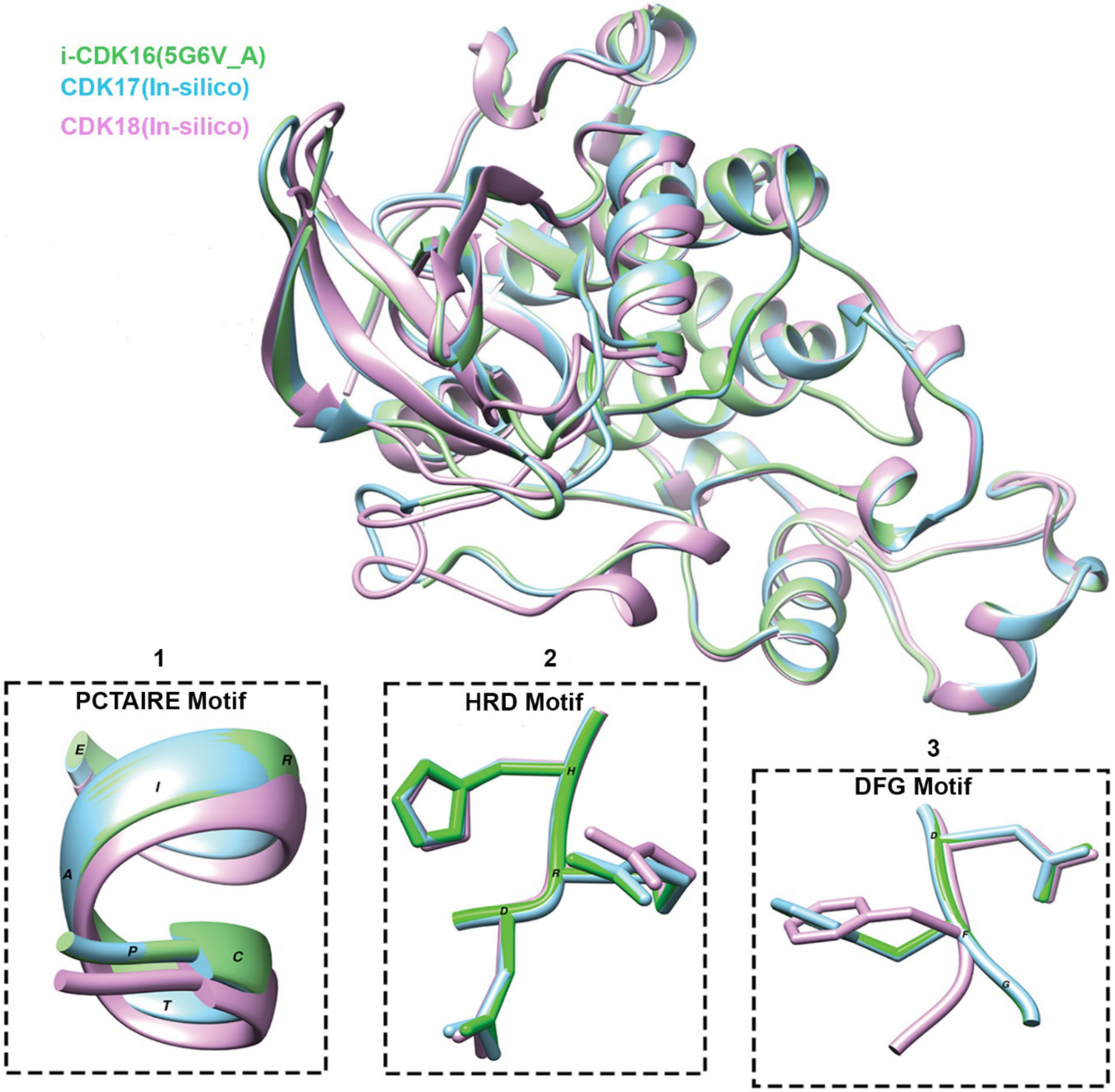

Supplementary Figure 7. Comparison of PCTAIRE 3D-structures. Structures of CDK17 and CDK18 were homology modelled using CDK16 [PDB-ID:5G6V] as template with SWISS-MODEL software.(10) Superimposition and visualization of structures was performed as described earlier (see footnote Supplementary Figure4). Generally, PCTAIREs are globally highly similar. In PCTAIRE motifs, marginal differences at 3D-coordinates are detectable (**inset 1**). The HRD motifs of *in silico* structures (CDK17 and CDK18) overlap very well with that of CDK16 (**inset 2**). In DFG motifs of PCTAIREs, slight deviation of the backbone, as well as of the side chain of phenylalanine of CDK18, is appreciable (**inset 3**).

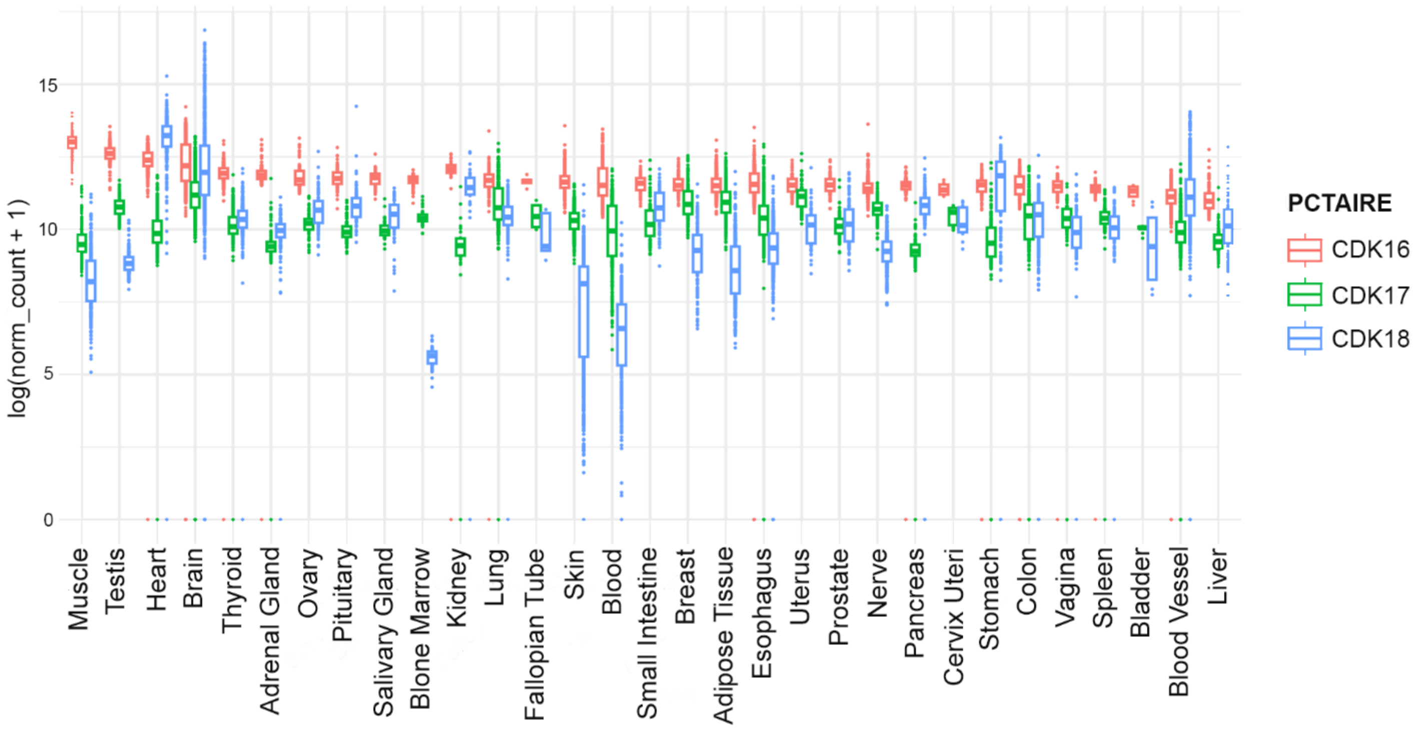

Supplementary Figure 8. **PCTAIREs expression in adult human tissues.** mRNA expression of PCTAIREs in human tissues were retrieved from Genotype-Tissue Expression (GTEx) Portal (11), visualized and plotted depicting the mean expression.

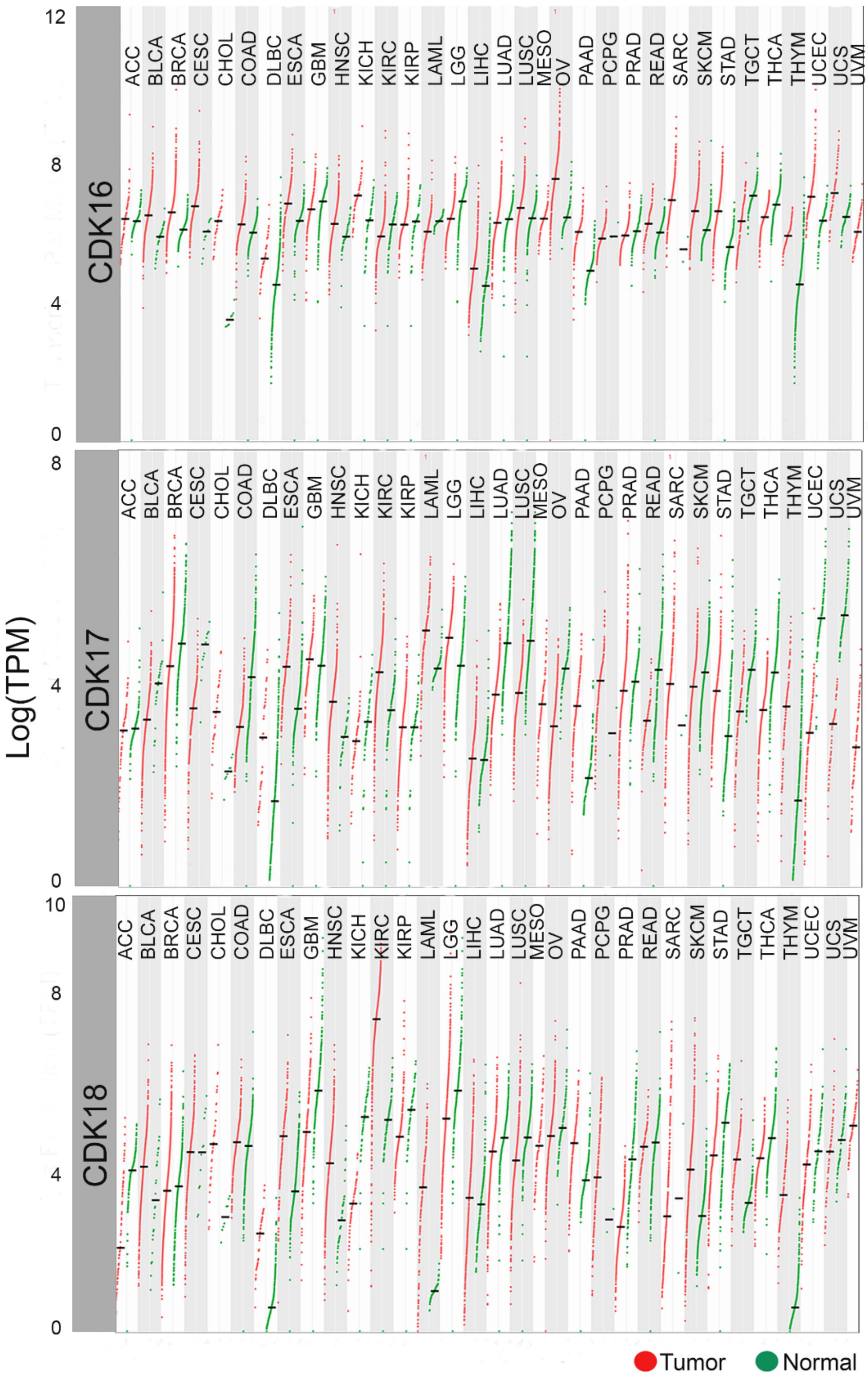

Supplementary Figure 9. PCTAIREs expression in tumours compared to normal matched counterpart. Graph reporting the mRNA expression levels of PCTAIREs across different cancer types and matched healthy tissues. Graphical representation was generated with Gepia webserver (12). TCGA datasets acronyms are specified in Supplementary Table 2.

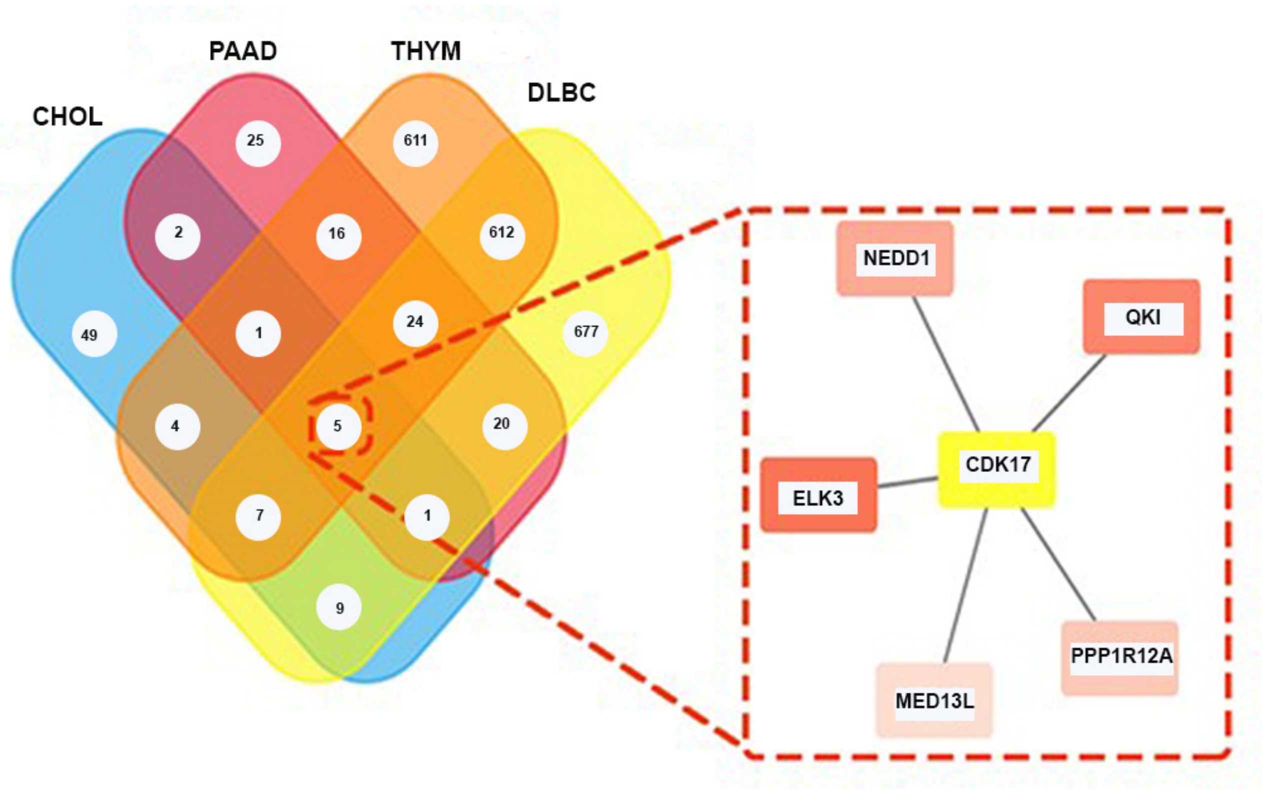

Supplementary Figure 10. **Putative CDK17 interactors in cancer.** Venn diagram shows genes that positively correlate with CDK17, at the mRNA expression level, in the indicated TGCA datasets (13). Only genes with a Spearman correlation coefficient > 6 are shown. Five genes, NEDD1, QKI, ELK3, MED13, and PPP1R12A, were shared among all datasets.

PCTAIRE Interactors								
CDK16			CDK17			CDK18		
Interactor	Co-IP	Ref.	Interactor	Co-IP	Ref.	Interactor	Co-IP	Ref.
CCNY	Endo. Exo.	27,32,35, 39,68	CABLES1	Exo.	41,82	CCNA2	Exo.	24
CCNYL1	Endo. Exo.	40	TRAP	Endo.	82	CCNE1	Exo.	24
CDK5/p35	Endo. Exo.	28				PKA	Exo.	24
14-3-3	Exo.	35,43,67						
**Tested PCTAIRE Substrates**								
MBP (Ref. 23,65)			Histone H1 (Ref. 42)			Rb (Ref. 24)		
**PCTAIRE Functions**								
Autophagy (Ref. 69)			Alzheimer's disease (Ref. 38)			Alzheimer's disease (Ref. 38,77,87)		
Cell proliferation (Ref. 36,98-100)			Autophagy (predicted) (Ref. 83)			Autophagy (predicted) (Ref. 83)		
Myogenesis (Ref. 79)			Glycerophospholipid Metabolism Pathway (predicted) (Ref. 80)			DNA Damage Repair (Ref. 126,128)		
Neurite Outgrowth (Ref. 23,70)			Mild Cognitive Impairment (predicted) (Ref. 77)			Nephrogenic Diabetes Insipidus (Ref. 37, 88-90)		
Regulation of microtubule network (Ref. 73)			Vesicular Transport (Ref. 71)			Type II Diabetes (predicted) (Ref. 92,93)		
Spermatogenesis (Ref. 27,40)			Viral Infection (predicted) (Ref. 81)			Vesicular Transport (Ref. 90)		
Vesicular Transport (Ref. 68,71)								

Supplementary Table 1. **Overview of PCTAIRE functions and interactome.** Table summarizes the most relevant PCTAIRE functions, substrates and interactors, subdivided for each family member (CDK16-CDK17-CDK18), based on available literature. PCTAIRE's interactors here reported were determined by CO-IP (Endogenous=Endo; Exogenous=Exo; MBP = Myelic Basic Protein; Rb = Retinoblastoma).

Acronym	TCGA Study name
LAML	Acute Myeloid Leukemia
ACC	Adrenocortical carcinoma
BLCA	Bladder Urothelial Carcinoma
LGG	Brain Lower Grade Glioma
BRCA	Breast invasive carcinoma
CESC	Cervical squamous cell carcinoma and endocervical adenocarcinoma
CHOL	Cholangiocarcinoma
COAD	Colon adenocarcinoma
ESCA	Esophageal carcinoma
GBM	Glioblastoma multiforme
HNSC	Head and Neck squamous cell carcinoma
KICH	Kidney Chromophobe carcinoma
KIRC	Kidney clear cell carcinoma
KIRP	Kidney papillary cell carcinoma
LIHC	Liver hepatocellular carcinoma
LUAD	Lung adenocarcinoma
LUSC	Lung squamous cell carcinoma
DLBC	Diffuse Large B-cell Lymphoma
MESO	Mesothelioma
OV	Ovarian serous cystadenocarcinoma
PAAD	Pancreatic adenocarcinoma
PCPG	Pheochromocytoma and Paraganglioma
PRAD	Prostate adenocarcinoma
READ	Rectum adenocarcinoma
SARC	Sarcoma
SKCM	Skin Cutaneous Melanoma
STAD	Stomach adenocarcinoma
TGCT	Testicular Germ Cell Tumors
THYM	Thymoma
THCA	Thyroid carcinoma
UCS	Uterine Carcinosarcoma
UCEC	Uterine Corpus Endometrial Carcinoma
UVM	Uveal Melanoma

Supplementary Table 2. TCGA datasets used in this study. Table summarizes the different datasets (with corresponding acronyms) used throughout the study.

Supplementary References

1. Zhou L, Feng T, Xu S, Gao F, Lam TT, Wang Q, et al. ggmsa: a visual exploration tool for multiple sequence alignment and associated data. Brief Bioinform. 2022 Jul 1;23(4):bbac222.

2. Charif D, Lobry JR. SeqinR 1.0-2: A Contributed Package to the R Project for Statistical Computing Devoted to Biological Sequences Retrieval and Analysis. In: Bastolla U, Porto M, Roman HE, Vendruscolo M, editors. Structural Approaches to Sequence Evolution: Molecules, Networks, Populations [Internet]. Berlin, Heidelberg: Springer Berlin Heidelberg; 2007. p. 207–32. Available from: https://doi.org/10.1007/978-3-540-35306-5_10

3. Gu Z, Eils R, Schlesner M. Complex heatmaps reveal patterns and correlations in multidimensional genomic data. Bioinformatics. 2016 Sep 15;32(18):2847–9.

4. Computation and biology: a partnership. Nat Methods. 2021 Jul 1;18(7):695–695.

5. Subramaniam S, Kleywegt GJ. A paradigm shift in structural biology. Nat Methods. 2022 Jan 1;19(1):20–3.

6. McSkimming DI, Rasheed K, Kannan N. Classifying kinase conformations using a machine learning approach. BMC Bioinformatics. 2017;18(1):86.

7. Dong R, Peng Z, Zhang Y, Yang J. mTM-align: an algorithm for fast and accurate multiple protein structure alignment. Bioinformatics. 2018 May 15;34(10):1719–25.

8. Meng EC, Pettersen EF, Couch GS, Huang CC, Ferrin TE. Tools for integrated sequence-structure analysis with UCSF Chimera. BMC Bioinformatics. 2006;7:1–10.

9. Dixon-Clarke SE, Shehata SN, Krojer T, Sharpe TD, von Delft F, Sakamoto K, et al. Structure and inhibitor specificity of the PCTAIRE-family kinase CDK16. Biochem J. 2017 Feb 20;474(5):699–713.

10. Waterhouse A, Bertoni M, Bienert S, Studer G, Tauriello G, Gumienny R, et al. SWISS-MODEL: Homology modelling of protein structures and complexes. Nucleic Acids Res. 2018;46(W1):W296–303.

11. GTEx Consortium. The Genotype-Tissue Expression (GTEx) project. Nat Genet. 2013 Jun;45(6):580–5.

12. Tang Z, Li C, Kang B, Gao G, Li C, Zhang Z. GEPIA: a web server for cancer and normal gene expression profiling and interactive analyses. Nucleic Acids Res. 2017 Jul 3;45(W1):W98–102.

13. Cerami E, Gao J, Dogrusoz U, Gross BE, Sumer SO, Aksoy BA, et al. The cBio Cancer Genomics Portal: An open platform for exploring multidimensional cancer genomics data. Cancer Discov. 2012;2(5):401–4.
